# Structural basis of nucleoside and nucleoside drug selectivity by concentrative nucleoside transporters

**DOI:** 10.7554/eLife.03604

**Published:** 2014-07-31

**Authors:** Zachary Lee Johnson, Jun-Ho Lee, Kiyoun Lee, Minhee Lee, Do-Yeon Kwon, Jiyong Hong, Seok-Yong Lee

**Affiliations:** 1 Department of Biochemistry, Duke University Medical Center, Durham, United States; 2 Department of Chemistry, Duke University, Durham, United States; 3 Department of Pharmacology and Cancer Biology, Duke University Medical Center, Durham, United States; Goethe University, Germany

**Keywords:** membrane transport, anticancer drugs, *Vibrio cholerae*, drug delivery, nucleoside, antiviral drugs, human, other

## Abstract

Concentrative nucleoside transporters (CNTs) are responsible for cellular entry of nucleosides, which serve as precursors to nucleic acids and act as signaling molecules. CNTs also play a crucial role in the uptake of nucleoside-derived drugs, including anticancer and antiviral agents. Understanding how CNTs recognize and import their substrates could not only lead to a better understanding of nucleoside-related biological processes but also the design of nucleoside-derived drugs that can better reach their targets. Here, we present a combination of X-ray crystallographic and equilibrium-binding studies probing the molecular origins of nucleoside and nucleoside drug selectivity of a CNT from *Vibrio cholerae*. We then used this information in chemically modifying an anticancer drug so that it is better transported by and selective for a single human CNT subtype. This work provides proof of principle for utilizing transporter structural and functional information for the design of compounds that enter cells more efficiently and selectively.

**DOI:** http://dx.doi.org/10.7554/eLife.03604.001

## eLife digest

DNA molecules are made from four bases—often named ‘G’, ‘A’, ‘C’, and ‘T’—that are arranged along a backbone made of sugars and phosphate groups. Chemicals called nucleosides are essentially the same as these four building blocks of DNA (and other similar molecules) but without the phosphate groups.

Proteins called nucleoside transporters are found in the membranes that surround cells and can pump nucleosides into the cell. These transporters also allow drugs that are made from modified nucleosides to enter cells; however, it was previously unclear how different transporters recognized and imported specific nucleosides.

Like other proteins, nucleoside transporters are basically strings of amino acids that have folded into a specific three-dimensional shape. A protein's shape is often important for defining what that protein can do, as often other molecules must bind to proteins—much like a key fitting into a lock. Johnson et al. have now revealed the three-dimensional structure of one nucleoside transporter protein bound to different nucleosides and nucleoside-derived chemicals, including three anti-cancer drugs and one anti-viral drug. Some of these chemicals were shown to bind more strongly to the transporter protein than others, and examining the three-dimensional structures revealed that the different chemicals interacted with slightly different amino acids in the transporter protein.

Johnson et al. then used this information to chemically modify an anticancer drug so that it is transported more easily into cells and is imported by only one of the subtypes of nucleoside transporters that are found in humans. This provides proof of principle that information about the structure and function of a transporter protein can help to redesign chemicals such that they can enter cells more efficiently, and to tailor them for transport by specific transporters. A similar approach may in the future allow researchers to design new nucleoside-derived drugs that are better at getting inside specific cells and, as such, provide effective treatments against cancers and viral infections.

**DOI:** http://dx.doi.org/10.7554/eLife.03604.002

## Introduction

Nucleosides play critical roles in biology as precursors to nucleic acids and the energy currency of the cell and also serve as signaling molecules (King et al., 2006; Rose and Coe, 2008; Molina-Arcas et al., 2009). Furthermore, nucleoside analogs have clinical applications as anticancer and antiviral drugs (Damaraju et al., 2003; Jordheim and Dumontet, 2007). Because of their immense biological and clinical importance, efficient entry of nucleosides and their analogs into the cell is crucial to human health and disease. Cellular entry is accomplished by a class of membrane proteins known as nucleoside transporters (NTs). There are two types of NTs in humans: concentrative nucleoside transporters (CNTs) and equilibrative nucleoside transporters (ENTs). CNTs utilize the energy of ion gradients to actively transport nucleosides into the cell against their concentration gradients while ENTs transport nucleosides down their chemical gradients without the requirement of any additional energy source (Gray et al., 2004b).

In addition to nucleosides, NTs are responsible for the transport of a wide range of nucleoside-derived anticancer (e.g., gemcitabine and 5-fluorouridine) and antiviral (e.g., ribavirin) drugs (Farre et al., 2004; Marechal et al., 2009; Rabascio et al., 2010; Bhutia et al., 2011; Doehring et al., 2011; Fukao et al., 2011; Rau et al., 2013). Both NT families possess subtype-dependent nucleoside specificities and tissue distributions, while CNTs are more highly subtype-specific for their substrates and distributions than ENTs (Gray et al., 2004b; Paproski et al., 2013). As a result, different NT subtypes are responsible for the transport of different types of nucleosides and nucleoside drugs, and expression levels of different NTs can predict how patients with certain types of cancer and viral infection will respond to nucleoside-drug treatment (Mackey et al., 1998; Farre et al., 2004; Spratlin et al., 2004; Gray et al., 2004a; Damaraju et al., 2009; Marechal et al., 2009; Rabascio et al., 2010; Bhutia et al., 2011; Doehring et al., 2011; Fukao et al., 2011; Rau et al., 2013). Since there are three different isoforms of human CNTs (hCNT1-3) that possess differing nucleoside and nucleoside-drug specificities and tissue distributions, greater knowledge of the molecular origins of nucleoside selectivity by CNTs could potentially lead to better-tailored nucleoside drug delivery as well as a better understanding of CNT-mediated physiological processes.

CNTs belong to the solute carrier (SLC) superfamily, constituting the family SLC28. The SLC superfamily, composed of 52 families, is responsible for the transport of ions, metabolites, neurotransmitters, and drugs in humans. Several SLC families are of particular clinical interest because of their roles in drug absorption, distribution, metabolism, and excretion (ADME) (Schlessinger et al., 2013). The recent determination of the structures of several SLC transporters has advanced our understanding of the inner workings of these transporters and expanded the applicability of structure-based ligand discovery using computational methods (Gao et al., 2009; He et al., 2010; Hu et al., 2011; Newstead et al., 2011; Johnson et al., 2012; Pedersen et al., 2013). Although this computational method of ligand discovery is a valuable approach, it cannot accurately predict the energetically important interactions between ligands and transporters, and therefore experimental approaches should be pursued to understand the principles of ligand and drug selectivity by these transporters.

The crystal structure of a CNT from *Vibrio cholerae* (vcCNT) presents the first opportunity to examine specific nucleoside recognition by CNTs from a structural perspective (Johnson et al., 2012). vcCNT is an excellent model system to study hCNTs: it utilizes a Na^+^ gradient for nucleoside transport like hCNTs and shares high sequence identity (36–39%) with hCNTs with particularly high sequence identity for the nucleoside-binding site (64% with hCNT1, 73% with hCNT2, 91% with hCNT3). For these reasons, vcCNT has been identified as an optimal candidate for structure-based ligand discovery using computational methods (Schlessinger et al., 2013).

Here, we have exploited a combination of X-ray crystallographic studies and equilibrium-binding measurements of vcCNT to understand the structural basis of CNT selectivity. We have discovered that CNTs use a unique mode of nucleoside recognition that is suitable for its function as a transporter. Using the insights gained from these studies, we have chemically modified the anticancer drug gemcitabine and found that its binding affinity for vcCNT is greatly enhanced. Furthermore, the modified compound now possesses subtype-specific transport among human CNTs. Follow-up structural and mutational studies revealed the origin of subtype-specificity of the modified compound. Not only do our studies illuminate the structural basis of nucleoside selectivity by CNTs but they also provide proof of principle for utilizing membrane transporter structures for the design of drugs with more selective delivery (Han and Amidon, 2000; Majumdar et al., 2004).

## Results

### The nucleoside-binding site of vcCNT-7C8C and equilibrium-binding measurements

**Figure 1.**
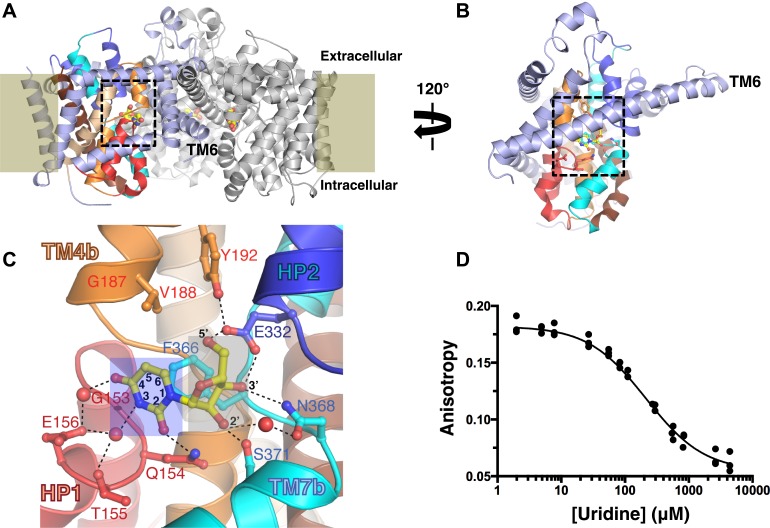
The nucleoside-binding site of vcCNT and fluorescence-anisotropy-based competition assay. (**A**) The vcCNT-7C8C trimer viewed from within the plane of the membrane. The location of the membrane is marked by rectangles. The scaffold domain of one protomer is colored light blue, and the transport domain is colored red, blue, orange, cyan, wheat, and brown. The other two protomers are colored gray. Uridine is shown bound to each protomer in stick representation. The nucleoside-binding site is delineated with dashed lines. vcCNT-7C8C functions similarly to wild type (Figure 1—figure supplement 1). (**B**) The vcCNT-7C8C protomer. The structure is rotated 120° about the trimer axis relative to **A**, zoomed in, and the other two protomers have been removed for clarity. (**C**) Nucleoside-binding site. Amino acid residues that interact with the uridine are labeled and shown in stick representation and were used for sequence identity calculation with hCNTs. Hydrogen bonds are shown as dashed lines. The uracil base is marked with a blue box, and the ribose is marked with a gray box. For a stereo view of the electron density in the nucleoside-binding site, see Figure 1—figure supplement 2. (**D**) Fluorescence titration of vcCNT with uridine. Uridine was titrated into solution containing vcCNT and the fluorescent nucleoside pyrrolo-cytidine, anisotropy was measured, and data were fit to a single-site competitive binding model to obtain a K_D_ of 36 ± 3 μM (mean ± SEM, n = 3 measurements). **DOI:** http://dx.doi.org/10.7554/eLife.03604.003

vcCNT forms a homotrimer with each protomer possessing its own nucleoside-binding site and permeation pathway (Figure 1A). The protomer adopts a new fold and is divided into two domains: the scaffold domain that is responsible for trimerization and maintaining the overall architecture of the transporter (light blue, Figure 1B), and the transport domain where nucleoside binding and transport occur (other colors, Figure 1B). The nucleoside-binding site, facing the trimer axis, is formed at the center of the transport domain between the tips of two helical hairpins (HP1 and HP2) and two partially unwound transmembrane helices (TM4 and TM7) (Johnson et al., 2012).

**Figure 1—figure supplement 1.**
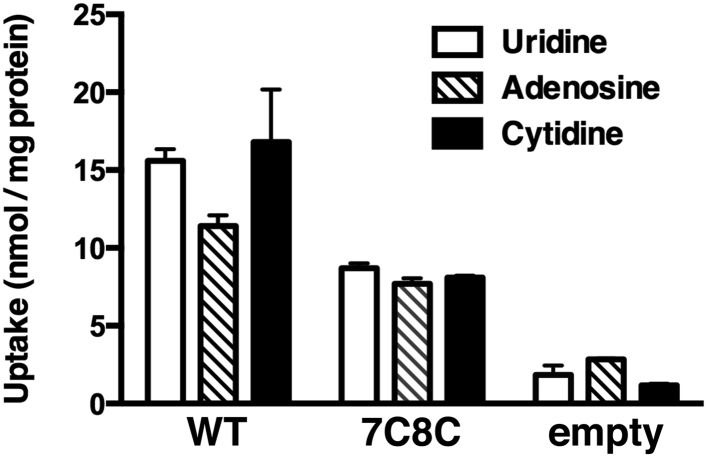
vcCNT-7C8C maintains nucleoside transport activity. Wild-type (WT) and 7C8C vcCNT were reconstituted into lipid vesicles and assessed for their ability to support the uptake of 2 μM of ^3^H-labeled nucleosides as described (Johnson et al., 2012). The data shown represent 2 min timepoints. Empty vesicles were included as a negative control. **DOI:** http://dx.doi.org/10.7554/eLife.03604.004

**Figure 1—figure supplement 2.**
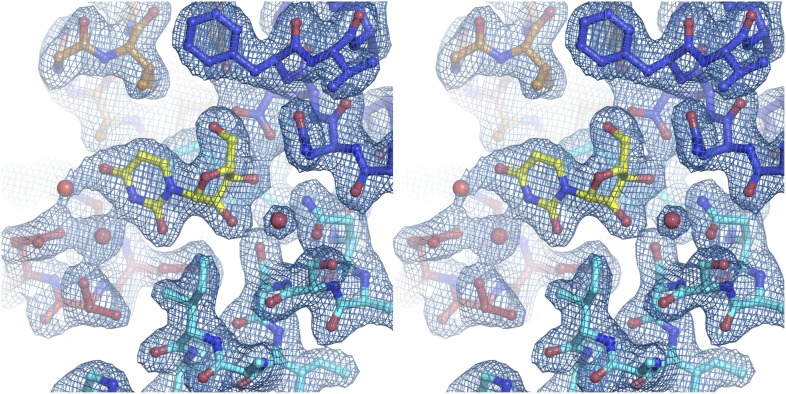
Electron density at the nucleoside-binding site of vcCNT-7C8C-uridine. The nucleoside-binding site of vcCNT-7C8C bound to uridine is shown in stereo in stick representation in the same orientation and colored as in Figure 1C. Uridine is yellow and the red spheres are water molecules. The resolution is 2.1 Å and density shown is from a 2F_o_–F_c_ electron density map contoured at 1σ. **DOI:** http://dx.doi.org/10.7554/eLife.03604.005

We solved the structure of a uridine-bound double mutant of vcCNT (Leu 7 to Cys and Ile 8 to Cys, termed 7C8C) at 2.1 Å, which is higher resolution than the 2.4-Å wild-type structure. This mutant was originally designed to introduce binding sites for hydrophobic mercury compounds for heavy-atom phasing. The high-resolution mutant structure revealed another water in the binding site that bridges the 4-carbonyl of the uracil base with Glu 156 but was otherwise identical to the wild-type structure (Figure 1C, Figure 1—figure supplement 2; Table 1). The mutations do not affect transporter function significantly (Figure 1—figure supplement 1).

**Table 1.** Data collection and refinement statistics **DOI:** http://dx.doi.org/10.7554/eLife.03604.006

	vcCNT-7C8C-uridine	vcCNT-zebularine	vcCNT-7C8C-adenosine	vcCNT-7C8C-ribavirin
Data collection				
Space group	P6_3_	P6_3_	P6_3_	P6_3_
Cell dimensions				
*a*, *b*, *c* (Å)	119.7, 119.7, 83.1	119.8, 119.8, 82.7	120.0, 120.0, 83.5	119.7, 119.7, 83.6
*α*, *β*, *γ* (°)	90, 90, 120	90, 90, 120	90, 90, 120	90, 90, 120
Resolution (Å)	2.08 (2.12–2.08)*	2.90 (2.95–2.90)	3.10 (3.15–3.10)	2.80 (2.85–2.80)
*R*_sym_ or *R*_merge_	0.052 (0.554)	0.141 (0.766)	0.104 (0.567)	0.114 (0.758)
*I*/σ*I*	42.0 (2.3)	13.5 (1.9)	14.1 (1.3)	20.2 (1.8)
Completeness (%)	99.3 (93.0)	99.9 (100.0)	99.0 (96.5)	99.8 (100.0)
Redundancy	5.9 (4.3)	5.8 (5.1)	4.2 (3.4)	7.0 (6.3)
Refinement				
Resolution (Å)	2.08 (2.13–2.08)	2.91 (3.13–2.91)	3.10 (3.41–3.10)	2.80 (2.98–2.80)
No. reflections	40368 (2381)	14932 (2798)	12429 (2887)	16840 (2635)
*R*_work_/*R*_free_ (%)	20.2/23.3	21.1/24.7	22.4/26.9	22.2/25.9
No. atoms				
Protein	2834	2868	2925	2947
Ligand/ion	17/1	16/1	19/1	17/1
Water/detergent	130/33	3/33	3/33	2/33
B-factors				
Protein	44.1	45.6	72.1	58.7
Ligand/ion	34.2/31.3	25.6/47.6	61.9/71.0	54.2/64.5
Water/detergent	54.4/62.6	29.3/56.4	54.6/89.0	48.1/72.3
R.m.s deviations				
Bond lengths (Å)	0.005	0.003	0.002	0.003
Bond angles (°)	0.782	0.676	0.617	0.709

	vcCNT-7C8C-5-fluorouridine	vcCNT-7C8C cytidine	vcCNT-7C8C-pyrrolo-cytidine	vcCNT-7C8C-gemcitabine
Data collection				
Space group	P6_3_	P6_3_	P6_3_	P6_3_
Cell dimensions				
*a*, *b*, *c* (Å)	119.8, 119.8, 83.2	120.0, 120.0, 82.5	119.6, 119.6, 83.1	119.0, 119.0, 82.3
*α*, *β*, *γ* (°)	90, 90, 120	90, 90, 120	90, 90, 120	90, 90, 120
Resolution (Å)	2.30 (2.34–2.30)	2.60 (2.64–2.60)	2.75 (2.80–2.75)	2.90 (2.97–2.90)
*R*_sym_ or *R*_merge_	0.067 (0.500)	0.094 (0.665)	0.083 (0.656)	0.061 (0.554)
*I*/σ*I*	28.0 (1.6)	24.0 (2.0)	22.6 (1.9)	30.8 (1.9)
Completeness (%)	97.3 (79.2)	99.3 (98.7)	99.9 (100.0)	99.6 (99.0)
Redundancy	4.0 (2.4)	6.4 (6.0)	5.8 (5.5)	5.7 (4.6)
Refinement				
Resolution (Å)	2.30 (2.38–2.30)	2.61 (2.75–2.61)	2.75 (2.92–2.75)	2.91 (3.13–2.91)
No. reflections	29380 (2089)	20600 (2754)	17698 (2784)	14682 (2751)
*R*_work/_ *R*_free_ (%)	20.0/21.6	21.4/23.9	20.2/24.7	22.7/25.8
No. atoms				
Protein	2937	2898	2921	2883
Ligand/ion	18/1	17/1	20/1	18/1
Water/detergent	58/33	28/33	3/33	9/33
B-factors				
Protein	54.4	59.1	60.9	76.7
Ligand/ion	41.4/47.2	46.1/48.6	48.4/54.1	59.6/70.1
Water/detergent	59.1/67.9	60.3/62.6	47.9/72.7	58.8/101.4
R.m.s deviations				
Bond lengths (Å)	0.002	0.002	0.003	0.004
Bond angles (°)	0.660	0.609	0.646	0.664

* Highest resolution shell is shown in parenthesis.

The structure of vcCNT bound to uridine revealed that the interactions can be divided into two groups: those that involve the ribose moiety and those with the nitrogenous base (Figure 1C). To determine the energetic contributions of each of the interactions between nucleoside and vcCNT, we developed a fluorescence-anisotropy-based competition assay for measuring the equilibrium dissociation constants (K_D_s) for a variety of nucleosides and nucleoside analogs using the fluorescent cytidine analog pyrrolo-cytidine (Table 2; Damaraju et al., 2011). We calculated the K_D_ for uridine to be 36 μM (Figure 1D), which is similar to the reported K_m_ values of uridine for hCNTs (K_m_ = 22–80 μM), further suggesting that vcCNT is a good model system to study hCNTs (Molina-Arcas et al., 2009).

**Table 2.** K_D_ values for nucleosides and nucleoside analog drugs calculated from fluorescence titrations **DOI:** http://dx.doi.org/10.7554/eLife.03604.007 10.7554/eLife.03604.008Table 2—source data 1.Fluorescence data for K_D_ calculations.For pyrrolo-cytidine and pyrrolo-gemcitabine, individual solutions with fixed nucleoside concentrations and increasing concentrations of vcCNT were prepared, the fluorescence anisotropy was measured, and the data were fit globally to a single-site binding model accounting for ligand depletion. For all other nucleosides, the nucleoside of interest was titrated into solution containing vcCNT and pyrrolo-cytidine 5 μl at a time, the fluorescence anisotropy was measured, and the data were fit globally to a single-site competitive binding model accounting for ligand depletion. All experiments were performed at least three times.**DOI:** http://dx.doi.org/10.7554/eLife.03604.008

Compound	K_D_ (μM)*
uridine	36 ± 3
cytidine	61 ± 5
adenosine	470 ± 100
gemcitabine	1,370 ± 430
ribavirin	1,530 ± 350
zebularine	120 ± 5
3-methyluridine	520 ± 80
5-fluorouridine	16 ± 1
5-chlorouridine	14 ± 1
5-iodouridine	58 ± 5
5-methyluridine	61 ± 7
2′-deoxyuridine	170 ± 10
3′-deoxyuridine	>2,800
5'-deoxyuridine	>2,800
cytarabine	>3,000
pyrrolo-cytidine	0.94 ± 0.17
pyrrolo-gemcitabine	23.5 ± 0.2

* Titrations were performed in triplicate and data were fit globally. All values are given as means ± SEM. See Table 2—source data 1 for fluorescence data used in calculating K_D_ values.

### Nucleobase interactions

Human CNTs have differing nucleoside-base preferences: hCNT1 mainly transports pyrimidines, hCNT2 prefers purines, and hCNT3 is broadly selective for both pyrimidines and purines (Gray et al., 2004b; Molina-Arcas et al., 2009). The uracil base interacts with residues on HP1 (Gln 154 directly and Thr 155 and Glu 156 through water molecules) and TM4 (Val 188 via van der Waals interactions). To examine the energetics of these interactions, we measured K_D_s of vcCNT for uridine analogs with modifications to the uracil base as well as other nucleosides.

**Figure 2.**
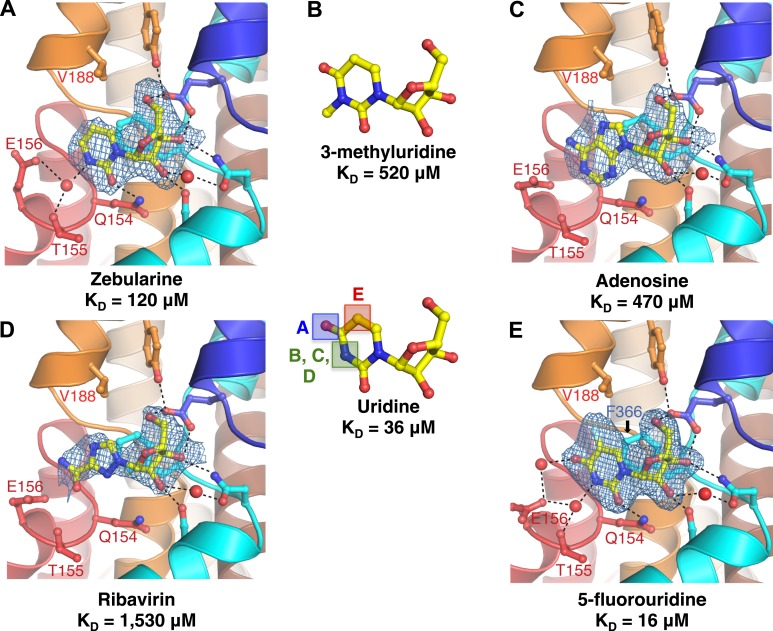
Structural basis of nucleobase recognition by vcCNT. (**A**) The crystal structure of vcCNT bound to zebularine. (**B**) Chemical structure of 3-methyluridine. (**C**) The crystal structure of vcCNT-7C8C bound to adenosine. (**D**) The crystal structure of vcCNT-7C8C bound to ribavirin. (**E**) The crystal structure of vcCNT-7C8C bound to 5-fluorouridine. Fluorine is colored cyan. All electron density maps represent F_o_–F_c_ SA-OMIT maps for the nucleoside contoured at 3σ. Uridine is shown in the center of the figure for reference. For stereo views of the electron density in the nucleoside-binding site for each of these structures, see Figure 2—figure supplements 1–4. **DOI:** http://dx.doi.org/10.7554/eLife.03604.009

The anticancer drug zebularine is a uridine analog with no substituent at the C4 position of the pyrimidine base. Zebularine exhibits a ∼threefold loss of binding affinity (K_D_ = 120 μM) relative to uridine. To deduce the structural basis of the reduced binding affinity, we solved the crystal structure of vcCNT bound to zebularine (Figure 2A, Figure 2—figure supplement 1). The crystal structure shows that the side chain of Glu 156 adopts a different rotamer position probably because it is unable to form the water-mediated interaction with the C4-carbonyl of the uracil base, consistent with the loss of binding affinity.

**Figure 2—figure supplement 1.**
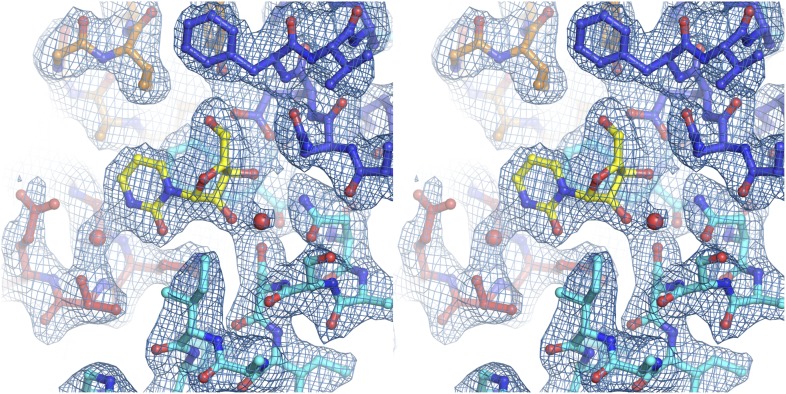
Electron density at the nucleoside-binding site of vcCNT-zebularine. The nucleoside-binding site of vcCNT bound to zebularine is shown in stereo in stick representation in the same orientation and colored as in Figure 2A. Zebularine is yellow and the red spheres are water molecules. The resolution is 2.9 Å and density shown is from a 2F_o_–F_c_ electron density map contoured at 1σ. **DOI:** http://dx.doi.org/10.7554/eLife.03604.010

**Figure 2—figure supplement 2.**
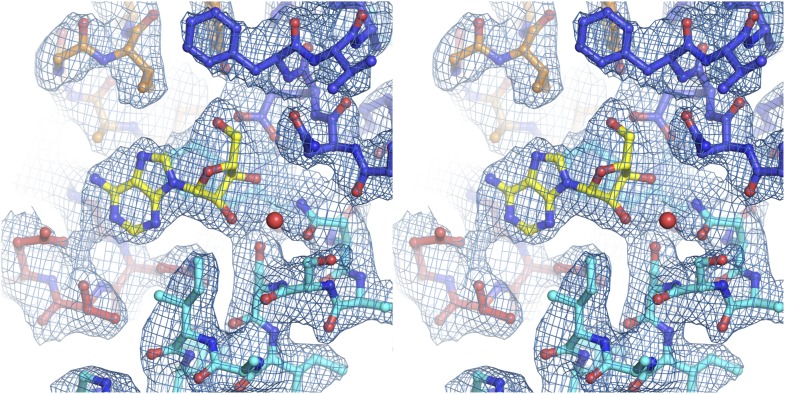
Electron density at the nucleoside-binding site of vcCNT-7C8C-adenosine. The nucleoside-binding site of vcCNT-7C8C bound to adenosine is shown in stereo in stick representation in the same orientation and colored as in Figure 2C. Adenosine is yellow and the red sphere is a water molecule. The resolution is 3.1 Å and density shown is from a 2F_o_–F_c_ electron density map contoured at 1σ. **DOI:** http://dx.doi.org/10.7554/eLife.03604.011

**Figure 2—figure supplement 3.**
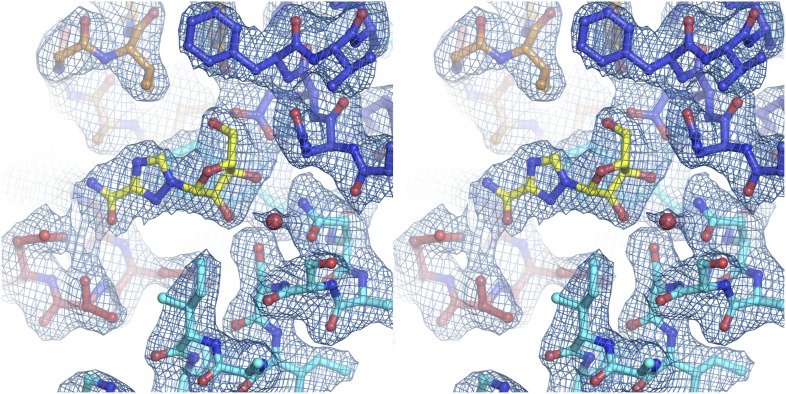
Electron density at the nucleoside-binding site of vcCNT-7C8C-ribavirin. The nucleoside-binding site of vcCNT-7C8C bound to ribavirin is shown in stereo in stick representation in the same orientation and colored as in Figure 2D. Ribavirin is yellow and the red sphere is a water molecule. The resolution is 2.8 Å and density shown is from a 2F_o_–F_c_ electron density map contoured at 1σ. **DOI:** http://dx.doi.org/10.7554/eLife.03604.012

**Figure 2—figure supplement 4.**
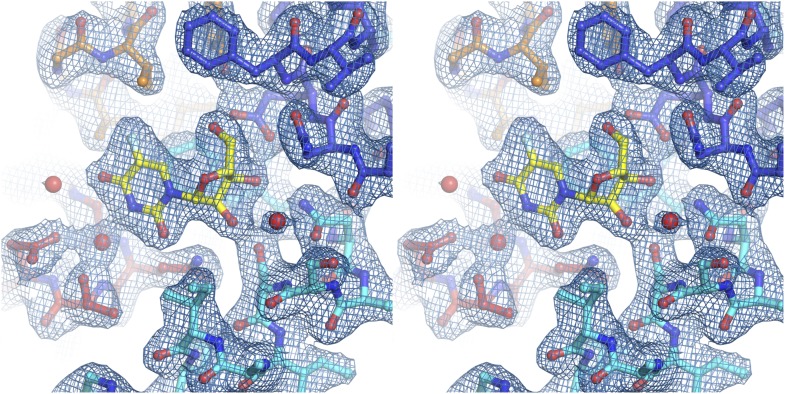
Electron density at the nucleoside-binding site of vcCNT-7C8C-5-fluorouridine. The nucleoside-binding site of vcCNT-7C8C bound to 5-fluorouridine is shown in stereo in stick representation in the same orientation and colored as in Figure 2E. 5-fluorouridine is yellow and the red spheres are water molecules. The resolution is 2.3 Å and density shown is from a 2F_o_–F_c_ electron density map contoured at 1σ. **DOI:** http://dx.doi.org/10.7554/eLife.03604.013

N3 of the uracil base interacts with both Thr 155 and Glu 156 through a single water molecule that is coordinated by both residues. We measured the affinity of vcCNT for 3-methyluridine, which contains a methyl group at this position that blocks the water-mediated interaction, and we found that it significantly decreased the binding affinity (K_D_ = 520 μM, Figure 2B, Figure 2—figure supplement 2). The purine nucleoside adenosine and the antiviral guanosine analog ribavirin also possess similarly weaker binding affinity (K_D_ = 470 and 1530 μM, respectively). To examine the structural basis of the reduced affinity, we solved the crystal structures of vcCNT-7C8C bound to adenosine (Figure 2C, Figure 2—figure supplement 3) and ribavirin (Figure 2D, Figure 2—figure supplement 4). The structures reveal that the bulky purine base displaces the water observed in the uridine structure while maintaining similar modes of ribose binding, corroborating the idea that the reduced affinities of adenosine and ribavirin are due to the loss of the water-mediated interactions and highlighting the importance of the water coordinated by Thr 155 and Glu 156 in nucleoside recognition by CNTs.

To test the energetic contribution of substituents at the C5 position of the pyrimidine ring, we measured the K_D_ for the anticancer drug 5-fluorouridine to be 16 μM, which is ∼twofold lower than the K_D_ for uridine. We also solved the crystal structure of vcCNT-7C8C bound to 5-fluorouridine (Figure 2E). The structure shows that the modified uracil base still fits into the nucleoside-binding site without any structural rearrangements of the protein. To understand the origin of enhanced affinity by the addition of fluorine at the C5 position, we compared the binding affinity of 5-fluorouridine to other C5-substituted uridine analogs with differing electronegativities and atomic radii (Table 3). Fluorine is highly electronegative (3.98 on the Pauling scale) and relatively small (1.47 Å radius). When another highly electronegative but slightly larger halogen, chlorine (3.16, 1.75 Å), is substituted at the C5 position, a similar K_D_ is observed (14 μM). However, when the large, weakly electronegative substituent iodine (2.66, 1.98 Å) or a methyl group (2.55, 2.00 Å) is added, we observe an increase in K_D_ (∼60 μM). In short, we observed an increase in affinity with smaller, highly electronegative substituents but a decrease in affinity with larger, less electronegative substituents. What structural feature could account for the differing affinities of these compounds?

**Table 3.** Properties of substituents of 5-substituted uridines and their binding affinities for vcCNT **DOI:** http://dx.doi.org/10.7554/eLife.03604.014

Substituent	Radius (Å)	Electronegativity	K_D_ (μM)
fluorine	1.47	3.98	16 ± 1
chlorine	1.75	3.16	14 ± 4
iodine	1.98	2.66	58 ± 5
methyl	2.00	2.55	61 ± 7

**Figure 3.**
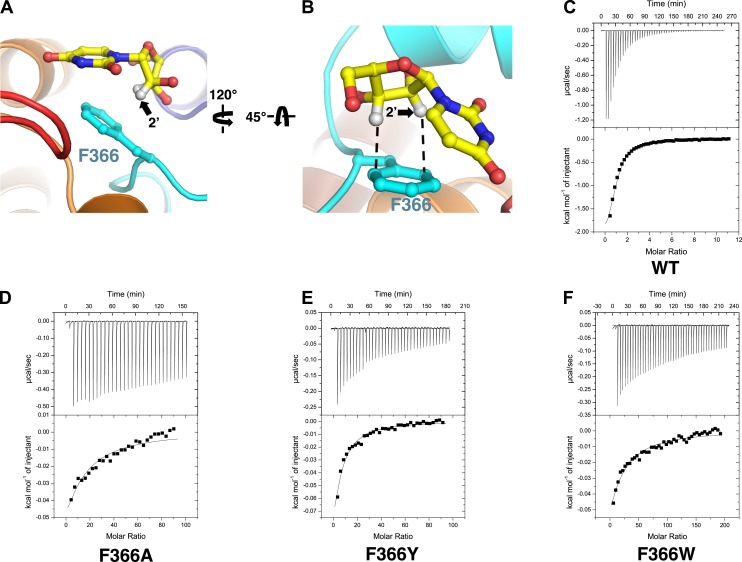
Phe 366 is crucial for nucleoside binding by vcCNT. (**A**) The nucleoside-binding site of vcCNT-7C8C bound to uridine is shown viewed from the cytoplasm. Phe 366 interacts with the uracil base via π–π interactions. The other epimeric 2′ position is marked with an arrow. (**B**) Another view of the interaction between Phe 366 and uridine. Phe 366 interacts with the ribose via CH–π interactions (dashed lines). (**C**–**F**) Isothermal titration calorimetry of uridine binding to wild-type vcCNT and Phe 366 mutants. K_D_ = 45 ± 8 μM and ΔH^o^ = −2970 ± 330 cal/mol for WT, K_D_ = 1630 ± 120 μM and ΔH^o^ = −2200 ± 190 cal/mol for F366A, K_D_ = 920 ± 170 μM and ΔH^o^ = −1600 ± 440 cal/mol for F366Y, and K_D_ = 1470 ± 90 μM and ΔH^o^ = −3190 ± 130 cal/mol for F366W (means ± SEM, n = 3 measurements). Note that the K_D_ for F366A could not be reliably measured due to the low heat associated with binding. Each of the F366 mutants is biochemically stable as evidenced by a single, sharp peak at the expected trimer size when subjected to size-exclusion chromatography (Figure 3—figure supplement 1). **DOI:** http://dx.doi.org/10.7554/eLife.03604.015

These differences may result from interactions of the nucleosides with Phe 366. In the structure of vcCNT-7C8C bound to uridine, Phe 366 appears to interact with both the uracil base and the ribose of the nucleoside. Phe 366 forms an offset π–π interaction with the aromatic pyrimidine ring and also forms CH–π interactions with the ribose (Figure 1C, Figure 3A,B). The addition of a small, highly electronegative substituent at the C5 position could strengthen the interaction between the pyrimidine ring and Phe 366, as is the case with many π–π interactions (Hunter and Sanders, 1990; Ringer et al., 2006).

**Figure 3—figure supplement 1.**
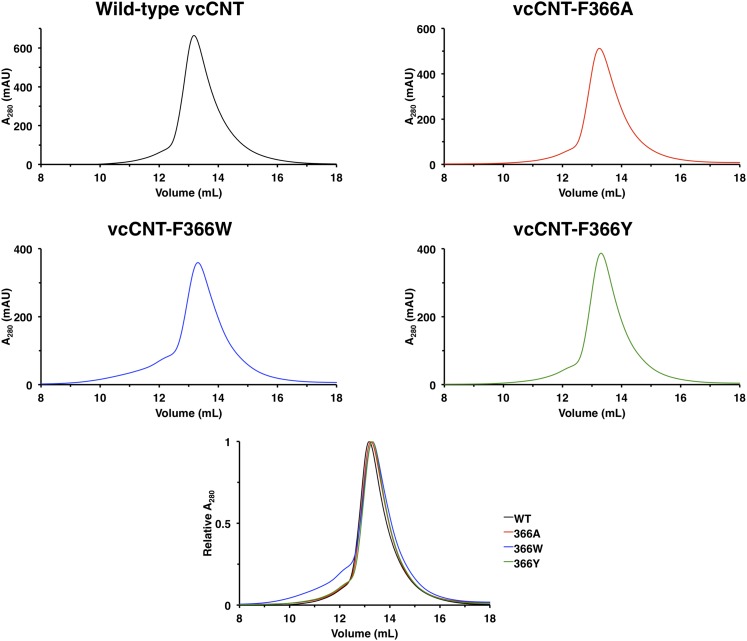
F366 mutants are biochemically stable. 1 liter of each construct was expressed and purified in parallel without the addition of any exogenous nucleosides as described in the ‘Materials and methods’. Each sample was initially purified by size-exclusion chromatography using a Superdex 200 10/300 GL column to remove MBP, and then the peak fractions were collected, concentrated, and re-ran to generate the figures. Each individual chromatogram is shown in the top panels with absolute absorbance at 280 nm on the y-axis, and all of the normalized chromatograms are shown overlaid in the bottom panel. **DOI:** http://dx.doi.org/10.7554/eLife.03604.016

Notably, Phe 366 is universally conserved between members of the CNT family, and its functional importance has never been tested. To examine the role of Phe 366 in nucleoside recognition, we mutated this residue and performed isothermal titration calorimetry (ITC) experiments. We found that the requirement for Phe at this position is strict, as mutation of this residue to alanine or tyrosine or tryptophan results in significantly decreased binding affinity for uridine while not affecting the stability significantly (Figure 3C–F, Figure 3—figure supplement 1). Therefore, we suggest that Phe 366 plays a critical role in recognition of the nucleoside by CNTs.

### Ribose interactions

**Figure 4.**
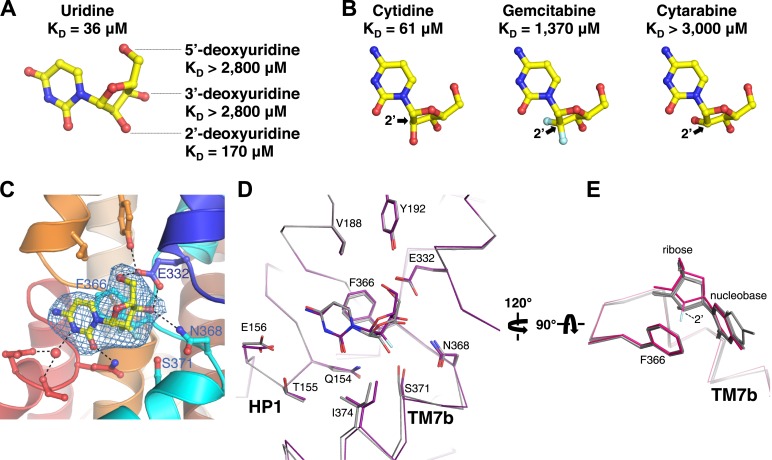
Structural basis of ribose recognition by vcCNT. (**A**) Dissociation constants for deoxyuridines. (**B**) Chemical structures and K_D_s of cytidine, gemcitabine, and cytarabine. The cytidine is from the crystal structure of vcCNT-7C8C bound to cytidine (for a stereo view of the electron density in the nucleoside-binding site of this structure, see Figure 4—figure supplement 1), and the other nucleosides are simply chemical structures in the same orientation as cytidine. Fluorine atoms in gemcitabine are colored cyan. (**C**) Crystal structure of vcCNT-7C8C bound to gemcitabine. Density shown is from an F_o_–F_c_ SA-OMIT map contoured at 3σ. For a stereo view of the electron density in the nucleoside-binding site, see Figure 4—figure supplement 2. (**D**) Alignment of uridine-bound and gemcitabine-bound vcCNT structures. Structures were aligned by Cα using PyMOL. TM7 was not used for the alignment. Cα traces and interacting amino acid residues are shown. The uridine-bound vcCNT structure (PDB ID: 3TIJ) is gray and the gemcitabine-bound vcCNT-7C8C structure is deep purple. (**E**) Alignment of vcCNT and vcCNT-7C8C structures bound to uridine (PDB ID: 3TIJ), zebularine, cytidine, pyrrolo-cytidine, 5-fluorouridine, and gemcitabine. Alignments were performed in the same manner as **D**. vcCNT-7C8C-gemcitabine is shown in hot pink and all other structures are shown in gray. **DOI:** http://dx.doi.org/10.7554/eLife.03604.017

Amino acid residues that interact with the ribose in the vcCNT structure (Glu 332, Asn 368, and Ser 371) are invariant between hCNTs and vcCNT. To probe these interactions, we measured K_D_s for deoxyuridines from which each of the ribose hydroxyls have been removed. Both 3′- and 5′-deoxyuridine yielded K_D_ values greater than 2800 μM (Figure 4A), while the binding affinity for 2′-deoxyuridine is not as drastically affected (K_D_ = 170 μM). Several anticancer nucleoside analog drugs contain modifications at the C2′ position of the ribose. Gemcitabine, for example, is a cytidine analog with two fluorine atoms bonded to C2′. The measured K_D_ of gemcitabine for vcCNT (K_D_ = 1370 μM) is ∼22-fold higher than that for cytidine (K_D_ = 61 μM) (Figure 4B, Figure 4—figure supplement 1). To understand the structural basis of this significant reduction in binding affinity associated with the fluorine substitutions, we solved the crystal structure of vcCNT-7C8C bound to gemcitabine (Figure 4C, Figure 4—figure supplement 2). In the uridine-bound structure, a CH–π interaction was observed between the C2′ hydrogen and Phe 366 (Figure 3B). In order for vcCNT to accommodate for the bulkier fluorine atom on the other epimeric position of C2′, which creates steric interference and electrostatic repulsion with the π electrons of Phe 366, both Phe 366 and TM7b (including Ser 371) move slightly away from the nucleoside with respect to the uridine-bound structure (Figure 4D). Furthermore, the ribose of the gemcitabine is reoriented with respect to the other nucleoside-bound structures (Figure 4E). In addition to the steric and electrostatic disruption of the ribose-binding site, fluorine is a poor substitute for a hydroxyl as a hydrogen-bond acceptor and thus provides a less favorable interaction of the ribose with Ser 371 (Howard et al., 1996; Dunitz and Taylor, 1997). To further test the importance of the epimeric position of the 2′-hydroxyl group of the ribose, we attempted to measure the K_D_ of another anticancer cytidine analog known as cytarabine (cytosine arabinoside) which has its 2′-hydroxyl flipped up above the ribose ring (Figure 4B). Cytarabine displayed no measurable binding when titrated into vcCNT (K_D_ > 3000 μM). Interestingly, consistent with our observation with vcCNT, hCNTs show no significant binding and transport of cytarabine (Clarke et al., 2006). Taken together, these results reveal that the interactions of both the nucleobase and the ribose with Phe 366 and the interactions of the ribose with TM7 are critical for nucleoside recognition by CNTs, which explains the intolerance of CNTs for substituents at the other epimeric position of C2′ of nucleosides.

**Figure 4—figure supplement 1.**
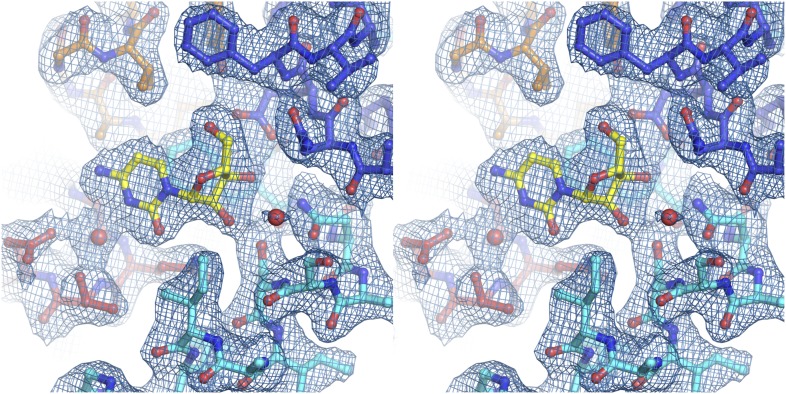
Electron density at the nucleoside-binding site of vcCNT-7C8C-cytidine. The nucleoside-binding site of vcCNT-7C8C bound to cytidine is shown in stereo in stick representation in the same orientation and colored as the structures in Figure 2. Cytidine is yellow and the red spheres are water molecules. The resolution is 2.6 Å and density shown is from a 2F_o_–F_c_ electron density map contoured at 1σ. **DOI:** http://dx.doi.org/10.7554/eLife.03604.018

**Figure 4—figure supplement 2.**
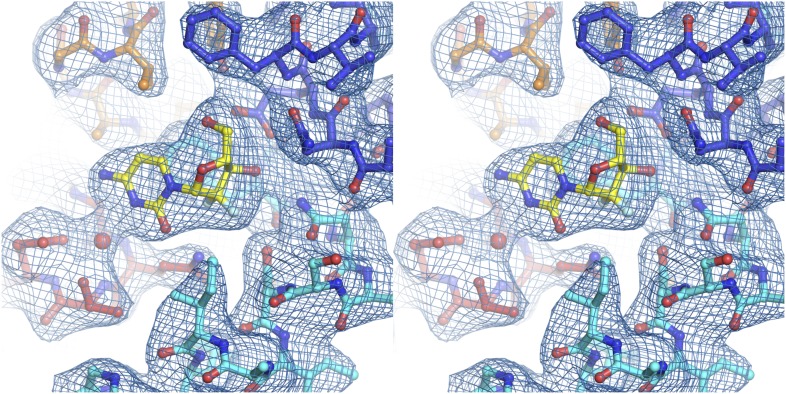
Electron density at the nucleoside-binding site of vcCNT-7C8C-gemcitabine. The nucleoside-binding site of vcCNT-7C8C bound to gemcitabine is shown in stereo in stick representation in the same orientation and colored as in Figure 4C. Gemcitabine is yellow and the red sphere is a water molecule. The resolution is 2.9 Å and density shown is from a 2F_o_–F_c_ electron density map contoured at 1σ. **DOI:** http://dx.doi.org/10.7554/eLife.03604.019

### Structure-based ligand modification

**Figure 5.**
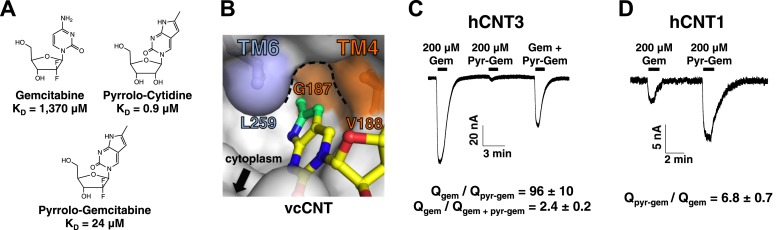
Design of pyrrolo-gemcitabine and its transportability by hCNTs. (**A**) Chemical structures and K_D_s of gemcitabine and the pyrrolo-nucleosides. (**B**) The crystal structure of vcCNT-7C8C in complex with pyrrolo-cytidine is shown in surface representation with pyrrolo-cytidine shown in stick representation. The additional three carbons that comprise the methylpyrrole ring of pyrrolo-cytidine are colored green. The vcCNT-7C8C nucleo-pocket, formed mainly by G187 (TM4), V188 (TM4), and L259 (TM6), is delineated with a dotted line. The location of the cytoplasm, adjacent to the nucleo-pocket, is shown. For a stereo view of the electron density in the nucleoside-binding site, see Figure 5—figure supplement 1. (**C**) hCNT3 transports gemcitabine but not pyrrolo-gemcitabine. Na^+^ currents were elicited by the addition of nucleoside to *Xenopus* oocytes expressing hCNT3, and currents were measured by two-electrode voltage-clamp. An example current trace is shown. For each individual oocyte, the area under each current peak was measured to calculate total charge (Q) transported during application of nucleoside. The ratio of total charge co-transported with gemcitabine to that with pyrrolo-gemcitabine or gemcitabine total charge to gemcitabine + pyrrolo-gemcitabine total charge was calculated for each oocyte experiment (means ± SEM, n = 14 oocytes). For Gem + Pyr-Gem, 200 μM of each nucleoside was added simultaneously. (**D**) hCNT1 transports pyrrolo-gemcitabine better than gemcitabine. Same experiment as in **C** but hCNT1-expressing oocytes were used and the ratio of total charge for pyrrolo-gemcitabine to gemcitabine is shown (means ± SEM, n = 11 oocytes). Neither gemcitabine nor pyrrolo-gemcitabine elicited currents in water-injected oocytes (Figure 5—figure supplement 2) See Figure 5—source data 1 for total charge source data. **DOI:** http://dx.doi.org/10.7554/eLife.03604.020 10.7554/eLife.03604.021Figure 5—source data 1.Total charge data.Total charge transported (μC) was calculated as the area under the curve of the current trace after application of the indicated nucleoside to hCNT-expressing *Xenopus* oocytes. Each row represents a separate experiment done using a different oocyte. The ratios of the total charge transported for the nucleosides are shown in the last columns.**DOI:** http://dx.doi.org/10.7554/eLife.03604.021

Of all of the nucleosides and nucleoside analogs studied, the fluorescence probe pyrrolo-cytidine had the strongest binding affinity for vcCNT (K_D_ = 0.9 μM, Figure 5A). We solved the crystal structure of vcCNT-7C8C bound to pyrrolo-cytidine and found that the methylpyrrole ring fits neatly into a pocket formed by TM4 and TM6 (Figure 5B, Figure 5—figure supplement 1). None of the other nucleosides in this study have moieties that can exploit this ‘nucleo-pocket’, and therefore this could be the root of the added strength of binding for pyrrolo-cytidine.

**Figure 5—figure supplement 1.**
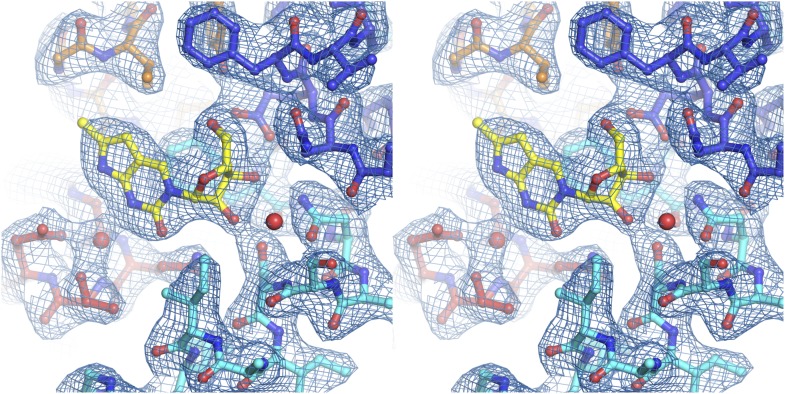
Electron density at the nucleoside-binding site of vcCNT-7C8C-pyrrolo-cytidine. The nucleoside-binding site of vcCNT-7C8C bound to pyrrolo-cytidine is shown in stereo in stick representation in the same orientation and colored as the structures in Figure 2. Pyrrolo-cytidine is yellow, and the red spheres are water molecules. The resolution is 2.8 Å and density shown is from a 2F_o_–F_c_ electron density map contoured at 1σ. **DOI:** http://dx.doi.org/10.7554/eLife.03604.022

**Figure 5—figure supplement 2.**
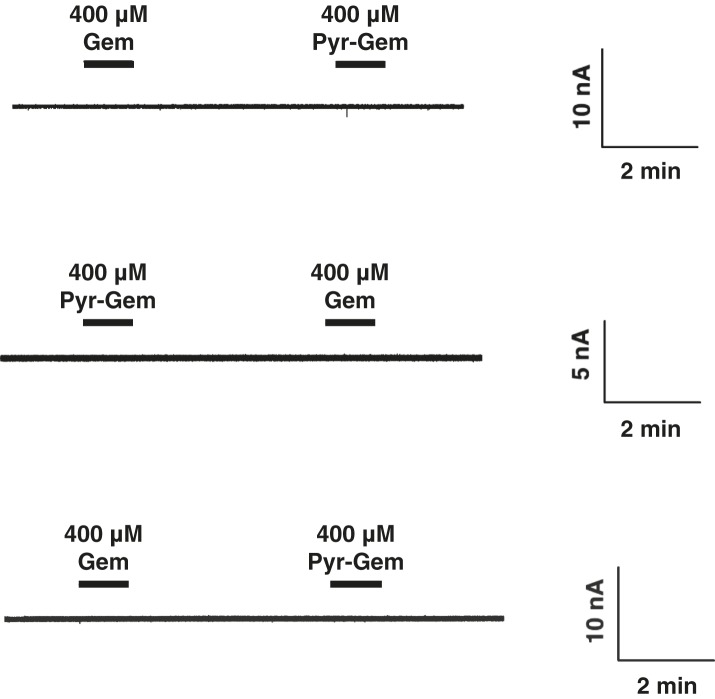
Water-injected oocytes do not respond to gemcitabine or pyrrolo-gemcitabine treatment. Current traces are shown for three different oocyte experiments. Experiments were performed in the same manner as those in 5C and 5D except water-injected oocytes were used. **DOI:** http://dx.doi.org/10.7554/eLife.03604.023

From our structural and binding studies, we learned that ribose interactions are important for CNT binding but nucleobase interactions are less stringent and can even be modified to improve binding. Because many nucleoside drugs contain modifications at the ribose (e.g., gemcitabine, AZT, and cytarabine), their apparent affinities for hCNTs are low (Graham et al., 2000; Clarke et al., 2006). We wondered whether this loss of affinity due to ribose modification could be compensated for by modification of the nucleobase. We synthesized a gemcitabine analog with the fluorescent nucleobase of pyrrolo-cytidine, which we now refer to as pyrrolo-gemcitabine (Figure 5A). We measured its binding affinity for vcCNT using the fluorescence-anisotropy assay and found that the K_D_ decreased by ∼60-fold to 24 μM, suggesting that nucleobase interactions and ribose interactions are additive.

We next sought to test how these results translated to transportability by hCNTs. It is known that hCNT1, hCNT3, and hENT1 are the main NTs that transport gemcitabine (Marechal et al., 2009; Bhutia et al., 2011; Damaraju et al., 2012; Paproski et al., 2013). To detect nucleoside transport by hCNTs, we turned to two-electrode voltage-clamp electrophysiological recording. Because hCNTs are Na^+^-nucleoside symporters, one can measure the current generated by the Na^+^ transport that is coupled with nucleoside transport. We injected *Xenopus* oocytes with mRNA coding for each of the hCNTs and measured inward Na^+^ currents elicited by the addition of different nucleosides to the extracellular side. Addition of 200 μM gemcitabine to hCNT3-expressing oocytes induced Na^+^ currents (Figure 5C, Figure 5—figure supplement 2). However, the same amount of pyrrolo-gemcitabine had almost no effect. The lack of Na^+^ current upon addition of pyrrolo-gemcitabine can either mean that hCNT3 is unable to bind the modified compound or hCNT3 binds but cannot transport the modified compound. To resolve this issue, a mixture of equal concentrations of both compounds was added and a reduction of Na^+^ current was observed, suggesting that hCNT3 binds to but does not transport pyrrolo-gemcitabine. In contrast, 200 μM pyrrolo-gemcitabine elicited ∼sevenfold higher total charge uptake than gemcitabine for hCNT1 (Figure 5D). hCNT2 transported neither compound (data not shown). Although both hCNT1 and hCNT3 can transport pyrimidines, by modifying the pyrimidine nucleobase of gemcitabine we have created a subtype-specific nucleoside analog with enhanced transportability by hCNT1.

**Figure 6.**
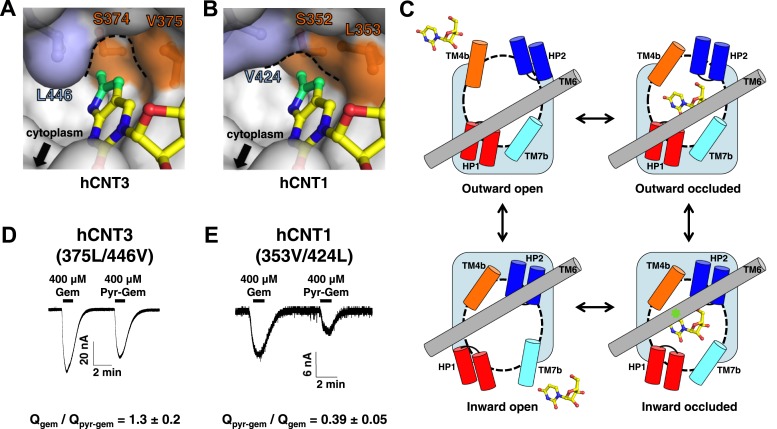
Structural basis of the subtype selectivity of pyrrolo-gemcitabine. (**A**) Model of hCNT3 nucleo-pocket. The structure of vcCNT-7C8C bound to pyrrolo-cytidine was used to generate a model of the hCNT3 nucleo-pocket by mutating the appropriate residues in PyMOL and selecting the rotamer that yielded the lowest amount of steric clash. (**B**) Model of hCNT1 nucleo-pocket. The model was generated in the same manner as **A**. Note that the methylpyrrole ring (green) will clash with the hCNT1 nucleo-pocket if it maintains the nucleoside-binding mode observed in the vcCNT structure. (**C**) Hypothetical alternating-access mechanism of vcCNT. A cartoon representation of the different conformational states along the transport cycle is depicted. The transport domain (including HP1, HP2, TM4b, and TM7b) and TM6 are shown as cylinders. Uridine is shown in stick representation. The nucleo-pocket in the inward-occluded conformation (bottom right) is located between TM6 and TM4 and is marked with a green star. The inward-occluded conformation is derived from the crystal structures of vcCNT. All other conformations are purely hypothetical. The transition between inward- and outward-facing conformations has been proposed to be achieved by a rigid-body movement of the transport domain across TM6 (Johnson et al., 2012). Extracellular and intracellular gating likely involves slight rearrangements of HP2/TM4b and HP1/TM7b, respectively. (**D**) hCNT3 (375L/446V) is capable of transporting both gemcitabine and pyrrolo-gemcitabine. Same experiment as Figure 5C,D but hCNT3 (375L/446V)-expressing oocytes were used and the ratio of total charge (Q) co-transported with gemcitabine to pyrrolo-gemcitabine is shown (means ± SEM, n = 11 oocytes). (**E**) hCNT1 (353V/424L) transports pyrrolo-gemcitabine less efficiently than gemcitabine (means ± SEM, n = 8 oocytes). Note that a higher nucleoside concentration was needed for **D** and **E** than the wild-type experiments due to lower transporter activity and/or expression. See Figure 6—source data 1 for total charge source data. **DOI:** http://dx.doi.org/10.7554/eLife.03604.024 10.7554/eLife.03604.025Figure 6—source data 1.Total charge data.Total charge transported (μC) was calculated as the area under the curve of the current trace after application of the indicated nucleoside to hCNT-expressing *Xenopus* oocytes. Each row represents a separate experiment done using a different oocyte. The ratios of the total charge transported for the nucleosides are shown in the last columns.**DOI:** http://dx.doi.org/10.7554/eLife.03604.025

The nucleo-pocket of vcCNT differs by only one amino acid (Gly 187 to Ser 374) from hCNT3 and three amino acids (Gly 187 to Ser 352, Val 188 to Leu 353, and Leu 259 to Val 424) from hCNT1. Since the nucleoside-binding site of vcCNT is highly homologous to hCNTs, we generated models of the hCNT1 and hCNT3 nucleo-pockets by swapping out these residues in the vcCNT-pyrrolo-cytidine structure. The structural models of hCNT1 and hCNT3 suggest changes in the overall structure of the nucleo-pocket (Figure 6A,B). In particular, the nucleo-pocket of the hCNT1 model does not have a large enough cavity to accommodate the pyrrole ring, and the opening to the intracellular solution is larger due to the smaller side chain on TM6. In the paradigm of the alternating-access mechanism of sodium-coupled symporters (Krishnamurthy et al., 2009), substrate release is achieved by the transition from the inward-facing occluded to the inward-facing open state (Figure 6C). Because the structure of vcCNT adopts an inward-facing occluded conformation where TM6, including Leu 259, serves as part of the gate (Johnson et al., 2012), the hCNT1 model suggests that the additional pyrrole group may destabilize the inward-occluded state and facilitate the transition to the inward-open state. In contrast, the additional pyrrole group may stabilize the inward-occluded state of hCNT3 and slow the transition into the inward-open state. Our hypothesis predicts that changing the structure of the nucleo-pocket in the inward-facing state would affect nucleoside transport by hCNTs. Consistent with our prediction, mutation of Val 375 and Leu 446 of hCNT3 to mimic the nucleo-pocket of hCNT1 leads to an increase in transport of pyrrolo-gemcitabine (Figure 6D). Furthermore, mutation of Leu 353 and Val 424 of hCNT1 to mimic the nucleo-pocket of hCNT3 leads to a decrease in transport of pyrrolo-gemcitabine (Figure 6E).

## Discussion

### Design principles of nucleoside recognition by CNTs

Our structural and equilibrium-binding studies of vcCNT have allowed us to better understand the design principles of nucleoside recognition by CNTs. Two helical hairpins (HP1 and HP2) and two unwound helices (TM4 and TM7), related by twofold pseudo-symmetry, create a bowl-shaped nucleoside-binding site at the center of the transport domain of vcCNT (Figure 1B,C). The interactions with the nucleobase are mainly formed with HP1 and TM4b, and the interactions with the ribose are mainly formed with HP2 and TM7b (Figure 1C). At the base of the bowl-shaped nucleoside-binding site, Phe 366 interacts with both the nucleobase and the ribose through π–π and CH–π interactions, respectively (Figure 3A,B). Three features make the architecture of the nucleoside-binding site of vcCNT particularly interesting: (1) the twofold pseudo-symmetry of the binding site that is divided in nucleobase and ribose binding on either side of the symmetry axis; (2) an aromatic ring at the base of the bowl that interacts with both the nucleobase and the ribose; (3) the localization of most of the protein–nucleoside interactions to one side of the nucleoside. These features of the nucleoside-binding site lead to the following questions: What are the energetics of the nucleobase and ribose interactions? What is the role of Phe 366? Why are the interactions with the nucleoside localized to the concave side of the bowl?

With regard to binding energetics, our equilibrium-binding studies have shown that the ribose interactions are energetically important consistent with previous non-equilibrium studies with hCNTs (Clarke et al., 2006). However, while disruption of the nucleobase interactions can have significant effects, the nucleobase can also be modified to improve binding. It is worth noting that our equilibrium-binding studies of vcCNT translate well to hCNT function. For example, we showed that the interaction between the C3′ ribose hydroxyl group and Ser 371 on TM7b is important in vcCNT (Figures 1C and 4A). Consistent with our observation, the hCNT1S546P variant is non-functional (Ser 546 in hCNT1 is equivalent to Ser 371 in vcCNT) (Cano-Soldado et al., 2012). Another important finding of our studies is that interactions with the nucleobase and ribose can be additive, and thus the loss of binding energy from modification of part of the nucleoside can be compensated for by the gain of energy from modification of another part of the nucleoside. Therefore, if a nucleoside drug contains a chemical modification necessary for its pharmacological function that hampers its recognition by CNTs, a compensatory chemical modification can be made so that the drug can still be recognized by CNTs.

Our structural and equilibrium-binding studies highlighted the importance of Phe 366 in nucleoside recognition. The nucleoside-bound structures of vcCNT help to shed light upon the structural basis of the importance of this residue. Notably, Phe 366 is the only residue within the nucleoside-binding site that forms interactions with both portions of the nucleoside. Furthermore, all of the other binding-site residues on HP1, HP2, TM4b, and TM7b interact from the same side of the nucleoside as Phe 366, forming the shape of a bowl around the nucleoside with Phe 366 serving as its base. As a result, the nucleoside rests on the face of Phe 366, likely helping to orient the nucleoside so that it may form all of the other interactions within the binding site. Although π–π and CH–π interactions are generally weak, they can provide significant interaction energies depending on the circumstance (Waters, 2002; Nishio, 2011). The importance of Phe 366 is demonstrated by the changes in affinity of C5-substituted uridine analogs for vcCNT by altering the π–π interaction with the nucleobase and the significant reduction of affinity of C2′-substituted nucleoside drugs via disruption of the CH–π interaction with the ribose. Furthermore, ITC experiments with Phe 366 mutants revealed the stringent requirement for phenylalanine at this position, as even replacement with tyrosine resulted in a significant loss of binding affinity for uridine. Taken together, we propose that the role of Phe 366 is to position the nucleoside for effective binding thus serving as a ‘selectivity ring’.

What is the molecular basis of having a bowl-shaped nucleoside-binding site? Several other nucleoside-binding proteins bind to their substrates by sandwiching the nucleobase between aromatic residues (Suzuki et al., 2004; Monecke et al., 2014). The utilization of a bowl-shaped binding site for a transporter makes practical sense as the substrate must be able to bind to and dissociate readily from the binding site in order for efficient transport to occur. We previously proposed that the transport domain undergoes a rigid-body motion while the scaffold domain, including TM6, remains static during the transition from the outward-occluded to the inward-occluded conformational state (Figure 6C; Johnson et al., 2012). In the inward-occluded conformation, TM6 (including Leu 259) is located on top of the bowl, serving as part of the intracellular gate and partially occluding the nucleoside from dissociating into the cytoplasm. Portions of HP1 and TM7b form the rest of the intracellular gate and may move slightly during the transition from the inward-occluded to the inward-open conformational state (Figure 6C), allowing the nucleoside to exit from the top of the bowl.

This rigid-body conformational change also suggests that most of the interactions between transporter and nucleoside that are present in the inward-occluded structure are maintained in the outward-facing conformation, as observed with other sodium-coupled transporters (Reyes et al., 2009; Zhou et al., 2014). One exception is the interaction between the methylpyrrole ring of pyrrolo-cytidine with the nucleo-pocket, which is likely to preferentially interact with the inward-occluded conformation because of the involvement of part of the scaffold domain (TM6, Figures 5B and 6C), which we expect to be immobile during the conformational change. While both conformations should be represented when the transporter is solubilized in detergent micelles, we anticipate that our structural and equilibrium-binding data revealed most of the amino acid residues that are important for nucleoside recognition. Consistent with our hypothesis, amino acid residues that were shown to be important from our inward-conformation-based binding studies in detergent have also been shown to be important for the binding and transport of nucleosides by human CNTs in several cell-based mutational studies (Loewen et al., 1999; Zhang et al., 2003, 2005; Yao et al., 2007; Slugoski et al., 2009). Further structural and biophysical studies probing the outward-facing state will help us to develop a complete understanding of the principles of nucleoside recognition by CNTs.

### Structure-based ligand modification and its implications

Despite the recent progress in the area of structural biology of transporters, most SLC transporter structures determined to date are not highly homologous to human transporters, and it is often the case that drug interactions with these non-human transporters are different from their human counterparts, rendering structural studies of drug–transporter interactions technically challenging and necessitating substantial engineering to mimic the behavior of the human transporters (Singh et al., 2007; Wang et al., 2013). Although our CNT is prokaryotic in origin, it is an excellent model system to study human CNTs and offers us the opportunity to conduct structural studies of nucleoside and nucleoside-drug selectivity by hCNTs.

Understanding the structural principles of nucleoside and nucleoside drug recognition by vcCNT not only allowed us to understand why a certain class of drugs (e.g., gemcitabine and cytarabine) are not well recognized and transported by hCNTs, but also offered us an opportunity to modify an existing drug to improve its affinity for vcCNT. Furthermore, our electrophysiological studies show that it is not only transported more efficiently by hCNT1, but it is also selectively transported by hCNT1. Our structural models of hCNT1 and hCNT3 allowed us to hypothesize that the subtype-specific differences in the structures of the nucleo-pocket in the inward-facing-occluded conformation give rise to the subtype-selectivity of the modified compound. The results of the mutational studies of the nucleo-pockets in hCNTs are consistent with this hypothesis. Although we do not know whether the modification of gemcitabine affects the outward conformation due to the lack of a structure of the outward-facing conformation, our studies suggest that destabilization of the inward-facing-occluded step would facilitate the release of the pyrrolo-gemcitabine. Conversely, stabilization of the outward-facing-occluded step would facilitate the capture of the substrate. Taken together, if one has knowledge of both the outward- and inward-facing conformations of the transporter, it might be possible, at least in principle, to modify a compound to bind to both conformations with differential affinities, which may improve its transportability and selectivity. Prior to our studies, the presence of the nucleo-pocket structure was unknown. Although our study with pyrrolo-gemcitabine serves merely as proof of concept, it is conceivable that the nucleo-pocket structure can be utilized in the design of nucleoside-derived drugs or prodrugs that can be specifically targeted only to cell types that express hCNT1 since expression levels of hCNT1 are closely related to the responsiveness of many different types of normal or cancer cells to chemotherapy treatment (Lane et al., 2010; Naito et al., 2010; Rabascio et al., 2010; Bhutia et al., 2011; Choi, 2012).

Finally, our studies provide another valuable concept: even though two transporter subtypes may share substrate and drug specificity, as is the case with hCNT1 and hCNT3, it is still possible to use structural differences (with the help of modeling) between the two subtypes to design or modify a drug that can be selectively transported. This concept has broad applications to many SLC transporters that are involved in ADME since many families such as SLC21, SLC22, and SLC29 possess subtype-dependent drug specificities and/or tissue distributions (Baldwin et al., 2004; Koepsell and Endou, 2004; Hagenbuch and Stieger, 2013).

These results can also have an impact on basic scientific research. Since some transporter subtypes show significant changes in expression levels between normal and pathological conditions (i.e., cancer), and these changes in transporter expression are usually important for the pathological conditions to persist, a fluorescent compound that is subtype-specific (e.g., pyrrolo-gemcitabine) for a certain transporter family can be a valuable tool to study the role of transporter subtypes in human health and disease through live cell imaging (Farre et al., 2004; Zhang et al., 2006; Bhutia et al., 2011; Perez-Torras et al., 2013). Taken together, this work not only represents an structural study of substrate and drug selectivity by membrane transporters, but our results also provide proof of principle for using this type of structure-function study for modifying drugs so that they are recognized and taken up into the cell by their cognate transporters more efficiently and selectively (Han and Amidon, 2000; Majumdar et al., 2004).

## Materials and methods

### Crystallization

Wild-type vcCNT and vcCNT-7C8C were expressed and purified as described (Johnson et al., 2012) in the absence of any added nucleoside. Briefly, protein was expressed as a His_10_-MBP fusion in C41 (DE3) cells, cells were lysed by homogenizer (AVESTIN, Ottawa, ON), protein was extracted from crude lysate using 30 mM dodecyl-maltoside, lysates were spun down to remove the insoluble fraction, and the supernatant was applied to a Co^2+^-affinity column for purification. The His_10_-MBP was cleaved by overnight digestion by PreScission Protease, and vcCNT was separated from His_10_-MBP by gel filtration using a Superdex 200 10/300 GL column in the presence of 5 mM decyl-maltoside. After gel filtration, protein was concentrated to ∼10 mg/ml and nucleoside was added to 1 mM (uridine), 2 mM (cytidine, adenosine), or 10 mM (ribavirin, gemcitabine, 5-fluorouridine, pyrrolo-cytidine, zebularine). Crystals were grown in the presence of 100 mM CaCl_2_, 37–42% PEG400, and 100 mM buffer: HEPES pH 7.5 (ribavirin), Tris–HCl pH 8.0–8.5 (adenosine, cytidine, gemcitabine, pyrrolo-cytidine), or glycine pH 9.5 (uridine, 5-fluorouridine, zebularine). Crystals were grown using the microbatch-under-oil technique. Crystals were harvested after 10–14 days, transferred to cryo solution containing 32.5% PEG400, and flash frozen in liquid nitrogen.

### Data collection and structure determination

X-ray data were collected at beamlines 22-ID-D and 24-ID-C at the Advanced Photon Source at Argonne National Laboratory. Data were processed using HKL-2000. The uridine, cytidine, zebularine, 5-fluorouridine, ribavirin, and pyrrolo-cytidine complex structures were refined using PHENIX with the original vcCNT structure (PDB ID 3TIJ) as the input model. The adenosine and gemcitabine complex structures were solved by molecular replacement with the original vcCNT structure as the search model using PHASER and refined using PHENIX.

### Fluorescence-based equilibrium-binding assay

To measure the binding affinity of vcCNT for fluorescent nucleoside analogs, individual 500-μl solutions were prepared containing varying concentrations of vcCNT in 5 mM DM and either 5 μM pyrrolo-cytidine or 2 μM pyrrolo-gemcitabine. The fluorescence anisotropy of each solution at λ_ex_ = 340 nm and λ_em_ = 467 nm was measured using a Cary Eclipse Fluorescence Spectrophotometer (Agilent, Santa Clara, CA) with automated polarizers. Each titration was performed at least three times. The data for the three titrations were simultaneously fit to a single-site binding model based off of Morrison's quadratic equation using nonlinear least-squares analysis in *GraphPad Prism* to obtain a dissociation constant and standard error.

To measure the binding affinity of vcCNT for other nucleosides and nucleoside analogs, the nucleoside of interest was titrated 5 μl at a time into 500 μl of solution initially containing 5 μM of vcCNT in 5 mM DM and 1–2 μM pyrrolo-cytidine. The fluorescence anisotropy after each addition was measured. Each titration was performed at least three times. The data for the three titrations were simultaneously fit to a one-site competitive binding model based off of Wang's method (Wang, 1995) using nonlinear least-squares analysis in *GraphPad Prism* to obtain a dissociation constant and standard error.

### Isothermal titration calorimetry

vcCNT mutants were prepared in the same manner as wild-type vcCNT. 15–30 mM of uridine was titrated 5 μl at a time into 25–40 μM of vcCNT solubilized in 5 mM DM using a MicroCal VP-ITC system (GE Healthcare, Pittsburgh, PA). The total heat exchanged during each injection was fit to a single-site binding isotherm with K_D_ and ΔH^o^ as independent parameters.

### Chemical synthesis

See Supplementary file 1 for a full description of the pyrrolo-gemcitabine synthesis. Briefly, the Sonogashira coupling of known 2′-deoxy-2′,2′-difluoro-5-iodo-uridine (Quintiliani et al., 2011) with propyne followed by Cu(I)-mediated cyclization provided the corresponding furano-gemcitabine in 64% for 2 steps. Treatment of furano-gemcitabine with NH_4_OH and CH_3_OH completed the synthesis of the desired pyrrolo-gemcitabine in 75%.

### *Xenopus laevis* oocyte expression and electrophysiology

The genes coding for the three hCNTs and vcCNT were cloned into a pGEM-HE vector. Plasmids were linearized using either SphI or NheI, and mRNA was transcribed using the mMESSAGE mMACHINE T7 Transcription Kit (Ambion, Grand Island, NY). Defolliculated *Xenopus laevis* oocytes were purchased from Ecocyte Bioscience (Austin, TX). Individual oocytes were injected with 40 ng of mRNA using a 10-μl microdispenser (Drummond Scientific, Broomall, PA) fitted with a tapered glass pipette tip and incubated at 17 °C for 4–5 days in ND96 buffer (96 mM NaCl, 2 mM KCl, 1 mM MgCl_2_, 1.8 mM CaCl_2_, and 5 mM HEPES pH 7.5) with 0.1% penicillin and streptomycin before recording.

The oocyte-recording chamber was gravity-perfused with ND96 buffer at a rate of 2 ml/min. Membrane currents were measured using an Oocyte Clamp (OC-725C; Warner Instruments, Hamden, CT). Individual oocytes were penetrated with two microelectrodes filled with 3 M KCl (0.5–1.0 MΩ). All electrophysiological experiments were conducted at room temperature. The OC-725C Oocyte Clamp was computer-interfaced via an Axon Digidata 1550 and controlled by Axoscope software (Molecular Devices, Sunnyvale, CA). The current signals were filtered at 20 Hz and sampled at intervals of 20 ms. The signals were filtered at 0.5 Hz by use of pCLAMP 10.4 software for data presentation. Ooctyes were impaled with the electrode filled with 3 M KCl, and then membrane potentials were observed for 10 min. Cells were discarded if resting membrane potentials were unstable or more positive than −30 mV. Oocyte membrane potentials were clamped at −90 mV for holding potentials to measure transporter-generated currents. All data are shown as means ± SEM.

## Additional files

Supplementary file 1. Synthetic approach to furano- and pyrrolo-gemcitabines. **DOI:** http://dx.doi.org/10.7554/eLife.03604.026

### Major datasets

The following datasets were generated:

JohnsonZL, LeeSY, 2014, Crystal structure of vcCNT-7C8C bound to ribavirin, http://www.rcsb.org/pdb/explore/explore.do?structureId=4PB1, Publicly available at RCSB Protein Data Bank.

JohnsonZL, LeeSY, 2014, Crystal structure of vcCNT-7C8C bound to 5-fluorouridine, http://www.rcsb.org/pdb/explore/explore.do?structureId=4PB2, Publicly available at RCSB Protein Data Bank.

JohnsonZL, LeeSY, 2014, Crystal structure of vcCNT-7C8C bound to gemcitabine, http://www.rcsb.org/pdb/explore/explore.do?structureId=4PD5, Publicly available at RCSB Protein Data Bank.

JohnsonZL, LeeSY, 2014, Crystal structure of vcCNT-7C8C bound to uridine, http://www.rcsb.org/pdb/explore/explore.do?structureId=4PD6, Publicly available at RCSB Protein Data Bank.

JohnsonZL, LeeSY, 2014, Crystal structure of vcCNT bound to zebularine, http://www.rcsb.org/pdb/explore/explore.do?structureId=4PD7, Publicly available at RCSB Protein Data Bank.

JohnsonZL, LeeSY, 2014, Crystal structure of vcCNT-7C8C bound to pyrrolo-cytidine, http://www.rcsb.org/pdb/explore/explore.do?structureId=4PD8, Publicly available at RCSB Protein Data Bank.

JohnsonZL, LeeSY, 2014, Crystal structure of vcCNT-7C8C bound to adenosine, http://www.rcsb.org/pdb/explore/explore.do?structureId=4PD9, Publicly available at RCSB Protein Data Bank.

JohnsonZL, LeeSY, 2014, Crystal structure of vcCNT-7C8C bound to cytidine, http://www.rcsb.org/pdb/explore/explore.do?structureId=4PDA, Publicly available at RCSB Protein Data Bank.

## References

[bib1] Baldwin SA Beal PR Yao SY King AE Cass CE Young JD 2004 The equilibrative nucleoside transporter family, SLC29. Pflugers Archiv 447:735–743. doi: 10.1007/s00424-003-1103-2 12838422

[bib2] Bhutia YD Hung SW Patel B Lovin D Govindarajan R 2011 CNT1 expression influences proliferation and chemosensitivity in drug-resistant pancreatic cancer cells. Cancer Research 71:1825–1835. doi: 10.1158/0008-5472.CAN-10-2736 21343396 PMC3048166

[bib3] Cano-Soldado P Gorraitz E Errasti-Murugarren E Casado FJ Lostao MP Pastor-Anglada M 2012 Functional analysis of the human concentrative nucleoside transporter-1 variant hCNT1S546P provides insight into the sodium-binding pocket. American Journal of Physiology Cell Physiology 302:C257–C266. doi: 10.1152/ajpcell.00198.2011 21998139

[bib4] Choi MK 2012 Variability of gemcitabine accumulation and its relationship to expression of nucleoside transporters in peripheral blood mononuclear cells. Archives of Pharmacal Research 35:921–927. doi: 10.1007/s12272-012-0518-8 22644860

[bib5] Clarke ML Damaraju VL Zhang J Mowles D Tackaberry T Lang T Smith KM Young JD Tomkinson B Cass CE 2006 The role of human nucleoside transporters in cellular uptake of 4'-thio-beta-D-arabinofuranosylcytosine and beta-D-arabinosylcytosine. Molecular Pharmacology 70:303–310. doi: 10.1124/mol.105.021543 16617163

[bib6] Damaraju VL Damaraju S Young JD Baldwin SA Mackey J Sawyer MB Cass CE 2003 Nucleoside anticancer drugs: the role of nucleoside transporters in resistance to cancer chemotherapy. Oncogene 22:7524–7536. doi: 10.1038/sj.onc.1206952 14576856

[bib7] Damaraju VL Mowles D Yao S Ng A Young JD Cass CE Tong Z 2012 Role of human nucleoside transporters in the uptake and cytotoxicity of azacitidine and decitabine. Nucleosides, Nucleotides & Nucleic Acids 31:236–255. doi: 10.1080/15257770.2011.652330 22356238

[bib8] Damaraju VL Sawyer MB Mackey JR Young JD Cass CE 2009 Human nucleoside transporters: biomarkers for response to nucleoside drugs. Nucleosides, Nucleotides & Nucleic Acids 28:450–463. doi: 10.1080/15257770903044499 20183595

[bib9] Damaraju VL Smith KM Mowles D Nowak I Karpinski E Young JD Robins MJ Cass CE 2011 Interaction of fused-pyrimidine nucleoside analogs with human concentrative nucleoside transporters: high-affinity inhibitors of human concentrative nucleoside transporter 1. Biochemical Pharmacology 81:82–90. doi: 10.1016/j.bcp.2010.09.009 20854794

[bib10] Doehring A Hofmann WP Schlecker C Zeuzem S Sarrazin C Berg T Muller T Herrmann E Geisslinger G Lotsch J 2011 Role of nucleoside transporters SLC28A2/3 and SLC29A1/2 genetics in ribavirin therapy: protection against anemia in patients with chronic hepatitis C. Pharmacogenetics and Genomics 21:289–296. doi: 10.1097/FPC.0b013e32834412e7 21346688

[bib11] Dunitz JD Taylor R 1997 Organic fluorine hardly ever accepts hydrogen bonds. Chemistry-A European Journal 3:89–98. doi: 10.1002/chem.19970030115

[bib12] Farre X Guillen-Gomez E Sanchez L Hardisson D Plaza Y Lloberas J Casado FJ Palacios J Pastor-Anglada M 2004 Expression of the nucleoside-derived drug transporters hCNT1, hENT1 and hENT2 in gynecologic tumors. International Journal of Cancer 112:959–966. doi: 10.1002/ijc.20524 15386342

[bib13] Fukao M Ishida K Sakamoto T Taguchi M Matsukura H Miyawaki T Hashimoto Y 2011 Effect of genetic polymorphisms of SLC28A1, ABCG2, and ABCC4 on bioavailability of mizoribine in healthy Japanese males. Drug Metabolism and Pharmacokinetics 26:538–543. doi: 10.2133/dmpk.DMPK-11-NT-040 21727755

[bib14] Gao X Lu F Zhou L Dang S Sun L Li X Wang J Shi Y 2009 Structure and mechanism of an amino acid antiporter. Science 324:1565–1568. doi: 10.1126/science.1173654 19478139

[bib15] Graham KA Leithoff J Coe IR Mowles D Mackey JR Young JD Cass CE 2000 Differential transport of cytosine-containing nucleosides by recombinant human concentrative nucleoside transporter protein hCNT1. Nucleosides, Nucleotides & Nucleic Acids 19:415–434. doi: 10.1080/15257770008033018 10772724

[bib16] Gray JH Mangravite LM Owen RP Urban TJ Chan W Carlson EJ Huang CC Kawamoto M Johns SJ Stryke D Ferrin TE Giacomini KM 2004a Functional and genetic diversity in the concentrative nucleoside transporter, CNT1, in human populations. Molecular Pharmacology 65:512–519. doi: 10.1124/mol.65.3.512 14978229

[bib17] Gray JH Owen RP Giacomini KM 2004b The concentrative nucleoside transporter family, SLC28. Pflugers Archiv 447:728–734. doi: 10.1007/s00424-003-1107-y 12856181

[bib18] Hagenbuch B Stieger B 2013 The SLCO (former SLC21) superfamily of transporters. Molecular Aspects of Medicine 34:396–412. doi: 10.1016/j.mam.2012.10.009 23506880 PMC3602805

[bib19] Han HK Amidon GL 2000 Targeted prodrug design to optimize drug delivery. AAPS PharmSci 2:E6 11741222 10.1208/ps020106 PMC2751001

[bib20] He X Szewczyk P Karyakin A Evin M Hong WX Zhang Q Chang G 2010 Structure of a cation-bound multidrug and toxic compound extrusion transporter. Nature 467:991–994. doi: 10.1038/nature09408 20861838 PMC3152480

[bib21] Howard JAK Hoy VJ Ohagan D Smith GT 1996 How good is fluorine as a hydrogen bond acceptor? Tetrahedron 52:12613–12622. doi: 10.1016/0040-4020(96)00749-1

[bib22] Hu NJ Iwata S Cameron AD Drew D 2011 Crystal structure of a bacterial homologue of the bile acid sodium symporter ASBT. Nature 478:408–411. doi: 10.1038/nature10450 21976025 PMC3198845

[bib23] Hunter CA Sanders JKM 1990 The Nature of pi-pi interactions. Journal of the American Chemical Society 112:5525–5534. doi: 10.1021/ja00170a016

[bib24] Johnson ZL Cheong CG Lee SY 2012 Crystal structure of a concentrative nucleoside transporter from *Vibrio cholerae* at 2.4 Å. Nature 483:489–493. doi: 10.1038/nature10882 22407322 PMC3310960

[bib25] Jordheim LP Dumontet C 2007 Review of recent studies on resistance to cytotoxic deoxynucleoside analogues. Biochimica et Biophysica Acta 1776:138–159. doi: 10.1016/j.bbcan.2007.07.004 17881132

[bib26] King AE Ackley MA Cass CE Young JD Baldwin SA 2006 Nucleoside transporters: from scavengers to novel therapeutic targets. Trends in Pharmacological Sciences 27:416–425. doi: 10.1016/j.tips.2006.06.004 16820221

[bib27] Koepsell H Endou H 2004 The SLC22 drug transporter family. Pflugers Archiv 447:666–676. doi: 10.1007/s00424-003-1089-9 12883891

[bib28] Krishnamurthy H Piscitelli CL Gouaux E 2009 Unlocking the molecular secrets of sodium-coupled transporters. Nature 459:347–355. doi: 10.1038/nature08143 19458710 PMC6821466

[bib29] Lane J Martin TA Mcguigan C Mason MD Jiang WG 2010 The differential expression of hCNT1 and hENT1 i n breast cancer and the possible impact on breast cancer therapy. Journal of Experimental Therapeutics & Oncology 8:203–210 20734919

[bib30] Loewen SK Ng AM Yao SY Cass CE Baldwin SA Young JD 1999 Identification of amino acid residues responsible for the pyrimidine and purine nucleoside specificities of human concentrative Na(+) nucleoside cotransporters hCNT1 and hCNT2. The Journal of Biological Chemistry 274:24475–24484. doi: 10.1074/jbc.274.35.24475 10455109

[bib31] Mackey JR Baldwin SA Young JD Cass CE 1998 Nucleoside transport and its significance for anticancer drug resistance. Drug Resistance Updates 1:310–324. doi: 10.1016/S1368-7646(98)80047-2 17092812

[bib32] Majumdar S Duvvuri S Mitra AK 2004 Membrane transporter/receptor-targeted prodrug design: strategies for human and veterinary drug development. Advanced Drug Delivery Reviews 56:1437–1452. doi: 10.1016/j.addr.2004.02.006 15191791

[bib33] Marechal R Mackey JR Lai R Demetter P Peeters M Polus M Cass CE Young J Salmon I Deviere J Van Laethem JL 2009 Human equilibrative nucleoside transporter 1 and human concentrative nucleoside transporter 3 predict survival after adjuvant gemcitabine therapy in resected pancreatic adenocarcinoma. Clinical Cancer Research 15:2913–2919. doi: 10.1158/1078-0432.CCR-08-2080 19318496

[bib34] Molina-Arcas M Casado FJ Pastor-Anglada M 2009 Nucleoside transporter proteins. Current Vascular Pharmacology 7:426–434. doi: 10.2174/157016109789043892 19485885

[bib35] Monecke T Buschmann J Neumann P Wahle E Ficner R 2014 Crystal structures of the novel cytosolic 5'-nucleotidase IIIB explain its preference for m7GMP. PLOS ONE 9:e90915. doi: 10.1371/journal.pone.0090915 24603684 PMC3946280

[bib36] Naito T Tokashiki S Mino Y Otsuka A Ozono S Kagawa Y Kawakami J 2010 Impact of concentrative nucleoside transporter 1 gene polymorphism on oral bioavailability of mizoribine in stable kidney transplant recipients. Basic & Clinical Pharmacology & Toxicology 106:310–316. doi: 10.1111/j.1742-7843.2009.00489.x 19912163

[bib37] Newstead S Drew D Cameron AD Postis VL Xia X Fowler PW Ingram JC Carpenter EP Sansom MS Mcpherson MJ Baldwin SA Iwata S 2011 Crystal structure of a prokaryotic homologue of the mammalian oligopeptide-proton symporters, PepT1 and PepT2. The EMBO Journal 30:417–426. doi: 10.1038/emboj.2010.309 21131908 PMC3025455

[bib38] Nishio M 2011 The CH/pi hydrogen bond in chemistry. Conformation, supramolecules, optical resolution and interactions involving carbohydrates. Physical Chemistry Chemical Physics 13:13873–13900. doi: 10.1039/c1cp20404a 21611676

[bib39] Paproski RJ Yao SY Favis N Evans D Young JD Cass CE Zemp RJ 2013 Human concentrative nucleoside transporter 3 transfection with ultrasound and microbubbles in nucleoside transport deficient HEK293 cells greatly increases gemcitabine uptake. PLOS ONE 8:e56423. doi: 10.1371/journal.pone.0056423 23441192 PMC3575408

[bib40] Pedersen BP Kumar H Waight AB Risenmay AJ Roe-Zurz Z Chau BH Schlessinger A Bonomi M Harries W Sali A Johri AK Stroud RM 2013 Crystal structure of a eukaryotic phosphate transporter. Nature 496:533–536. doi: 10.1038/nature12042 23542591 PMC3678552

[bib41] Perez-Torras S Vidal-Pla A Cano-Soldado P Huber-Ruano I Mazo A Pastor-Anglada M 2013 Concentrative nucleoside transporter 1 (hCNT1) promotes phenotypic changes relevant to tumor biology in a translocation-independent manner. Cell Death & Disease 4:e648. doi: 10.1038/cddis.2013.173 23722537 PMC3674379

[bib42] Quintiliani M Persoons L Solaroli N Karlsson A Andrei G Snoeck R Balzarini J Mcguigan C 2011 Design, synthesis and biological evaluation of 2'-deoxy-2',2'-difluoro-5-halouridine phosphoramidate ProTides. Bioorganic & Medicinal Chemistry 19:4338–4345. doi: 10.1016/j.bmc.2011.05.037 21696963 PMC7127735

[bib43] Rabascio C Laszlo D Andreola G Saronni L Radice D Rigacci L Fabbri A Frigeri F Calabrese L Billio A Bertolini F Martinelli G 2010 Expression of the human concentrative nucleotide transporter 1 (hCNT1) gene correlates with clinical response in patients affected by Waldenstrom's Macroglobulinemia (WM) and small lymphocytic lymphoma (SLL) undergoing a combination treatment with 2-chloro-2'-deoxyadenosine (2-CdA) and Rituximab. Leukemia Research 34:454–457. doi: 10.1016/j.leukres.2009.07.002 19647871

[bib44] Rau M Stickel F Russmann S Manser CN Becker PP Weisskopf M Schmitt J Dill MT Dufour JF Moradpour D Semela D Mullhaupt B Geier A & Swiss Hepatitis, CCSG 2013 Impact of genetic SLC28 transporter and ITPA variants on ribavirin serum level, hemoglobin drop and therapeutic response in patients with HCV infection. Journal of Hepatology 58:669–675. doi: 10.1016/j.jhep.2012.11.027 23195617

[bib45] Reyes N Ginter C Boudker O 2009 Transport mechanism of a bacterial homologue of glutamate transporters. Nature 462:880–885. doi: 10.1038/nature08616 19924125 PMC2934767

[bib46] Ringer AL Sinnokrot MO Lively RP Sherrill CD 2006 The effect of multiple substituents on sandwich and T-shaped pi-pi interactions. Chemistry: A European Journal 12:3821–3828. doi: 10.1002/chem.200501316 16514687

[bib47] Rose JB Coe IR 2008 Physiology of nucleoside transporters: back to the future. Physiology 23:41–48. doi: 10.1152/physiol.00036.2007 18268364

[bib48] Schlessinger A Yee SW Sali A Giacomini KM 2013 SLC classification: an update. Clinical Pharmacology and Therapeutics 94:19–23. doi: 10.1038/clpt.2013.73 23778706 PMC4068830

[bib49] Singh SK Yamashita A Gouaux E 2007 Antidepressant binding site in a bacterial homologue of neurotransmitter transporters. Nature 448:952–956. doi: 10.1038/nature06038 17687333

[bib50] Slugoski MD Smith KM Ng AM Yao SY Karpinski E Cass CE Baldwin SA Young JD 2009 Conserved glutamate residues Glu-343 and Glu-519 provide mechanistic insights into cation/nucleoside cotransport by human concentrative nucleoside transporter hCNT3. The Journal of Biological Chemistry 284:17266–17280. doi: 10.1074/jbc.M109.009613 19380587 PMC2719363

[bib51] Spratlin J Sangha R Glubrecht D Dabbagh L Young JD Dumontet C Cass C Lai R Mackey JR 2004 The absence of human equilibrative nucleoside transporter 1 is associated with reduced survival in patients with gemcitabine-treated pancreas adenocarcinoma. Clinical Cancer Research 10:6956–6961. doi: 10.1158/1078-0432.CCR-04-0224 15501974

[bib52] Suzuki NN Koizumi K Fukushima M Matsuda A Inagaki F 2004 Structural basis for the specificity, catalysis, and regulation of human uridine-cytidine kinase. Structure 12:751–764. doi: 10.1016/j.str.2004.02.038 15130468

[bib53] Wang H Goehring A Wang KH Penmatsa A Ressler R Gouaux E 2013 Structural basis for action by diverse antidepressants on biogenic amine transporters. Nature 503:141–145. doi: 10.1038/nature12648 24121440 PMC3904662

[bib54] Wang ZX 1995 An exact mathematical expression for describing competitive binding of two different ligands to a protein molecule. FEBS Letters 360:111–114. doi: 10.1016/0014-5793(95)00062-E 7875313

[bib55] Waters ML 2002 Aromatic interactions in model systems. Current Opinion in Chemical Biology 6:736–741. doi: 10.1016/S1367-5931(02)00359-9 12470725

[bib56] Yao SY Ng AM Slugoski MD Smith KM Mulinta R Karpinski E Cass CE Baldwin SA Young JD 2007 Conserved glutamate residues are critically involved in Na+/nucleoside cotransport by human concentrative nucleoside transporter 1 (hCNT1). The Journal of Biological Chemistry 282:30607–30617. doi: 10.1074/jbc.M703285200 17704058

[bib57] Zhang J Smith KM Tackaberry T Visser F Robins MJ Nielsen LP Nowak I Karpinski E Baldwin SA Young JD Cass CE 2005 Uridine binding and transportability determinants of human concentrative nucleoside transporters. Molecular Pharmacology 68:830–839 15955867 10.1124/mol.105.012187

[bib58] Zhang J Sun X Smith KM Visser F Carpenter P Barron G Peng Y Robins MJ Baldwin SA Young JD Cass CE 2006 Studies of nucleoside transporters using novel autofluorescent nucleoside probes. Biochemistry 45:1087–1098. doi: 10.1021/bi0520535 16430205

[bib59] Zhang J Visser F Vickers MF Lang T Robins MJ Nielsen LP Nowak I Baldwin SA Young JD Cass CE 2003 Uridine binding motifs of human concentrative nucleoside transporters 1 and 3 produced in *Saccharomyces cerevisiae*. Molecular Pharmacology 64:1512–1520. doi: 10.1124/mol.64.6.1512 14645682

[bib60] Zhou X Levin EJ Pan Y Mccoy JG Sharma R Kloss B Bruni R Quick M Zhou M 2014 Structural basis of the alternating-access mechanism in a bile acid transporter. Nature 505:569–573. doi: 10.1038/nature12811 24317697 PMC4142352

