# Supplementary material for: Structural basis of nucleoside and nucleoside drug selectivity by concentrative nucleoside transporters
Source: eLife. 2014 Jul 31;3:e03604. doi: 10.7554/eLife.03604 (PMC4139061; doi:10.7554/eLife.03604)
Supplement: Supplementary file 1. — Synthetic approach to furano- and pyrrolo-gemcitabines. DOI: http://dx.doi.org/10.7554/eLife.03604.026 [file elife03604s004.pdf]

Supplementary file 1

Synthetic Approach to Furano- and Pyrrolo-Gemcitabines

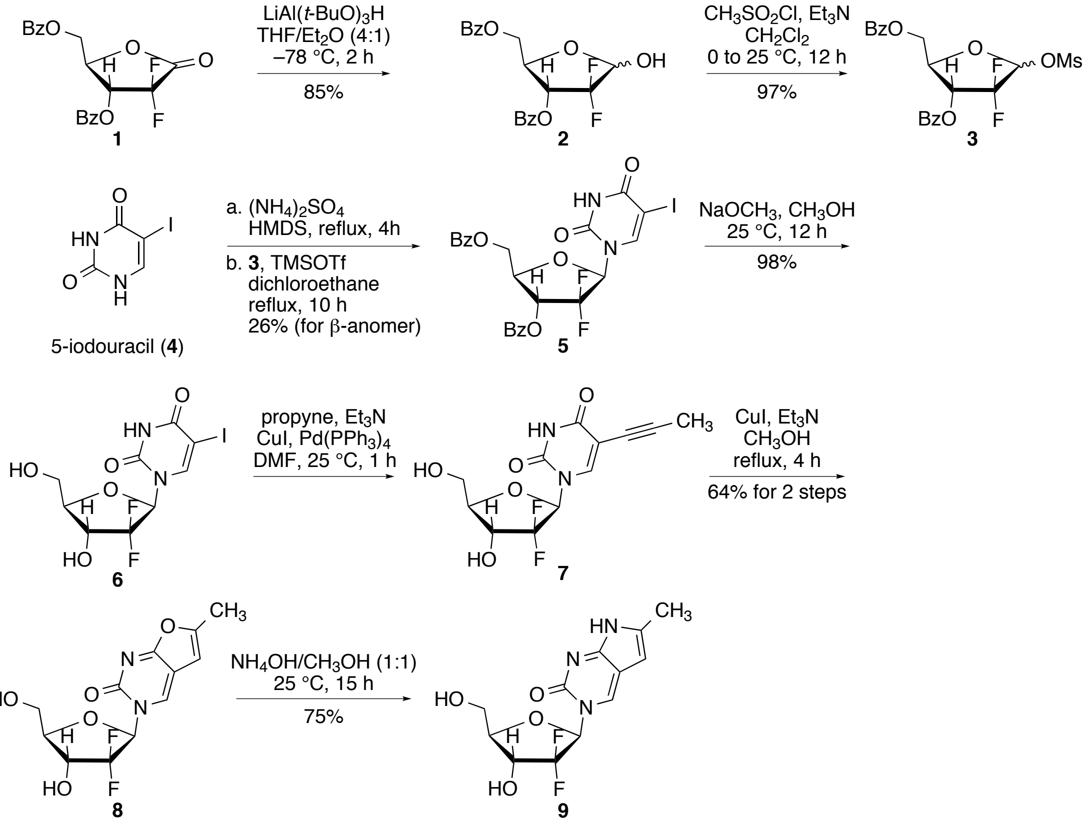

Preparation of Furano-Gemcitabine 8

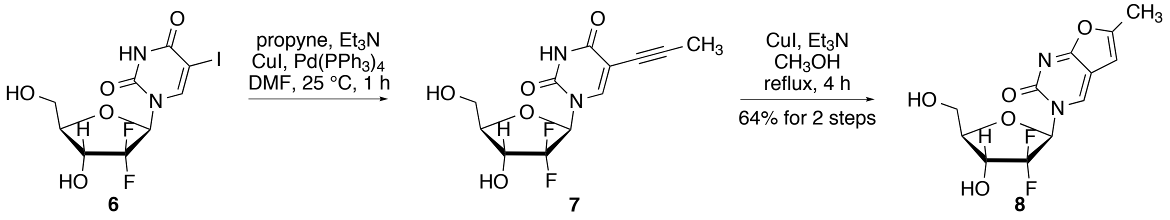

[**Sonogashira Coupling**] To a solution of known 2’-deoxy-2’,2’-difluoro-5-iodo-uridine **6** (100 mg, 0.256 mmol) in DMF (3 mL, 0.085 M) were added CuI (12 mg, 0.062 mmol), Et_3N (71 μL, 0.512 mmol), and $Pd(PPh_3)_4$ (36.2 mg, 0.031 mmol) [30]. The resulting mixture was treated with propyne (150 mg, 3.753 mmol) and stirred for 1 h at 25 ºC. The reaction mixture was filtered through a pad of silica

gel ($CHCl_3$/MeOH = 9/1) and concentrated *in vacuo*. The crude alkyne **7** was employed in the next step without further purification.

[**Cyclization**] To a stirred solution of **7** (55 mg, 0.182mmol) in Et_3N/MeOH (3:7, 3 mL) was added CuI (5 mg, 0.026 mmol). After stirring for 4 h at reflux, the resulting mixture was concentrated *in vacuo*. The residue was purified by column chromatography (SiO_2, $CHCl_3$/MeOH = 8/1) to afford furano-gemcitabine **8** (50 mg, 64% for 2 steps) as a brown solid: ^{1}H NMR (400 MHz, DMSO-d_6) δ 8.57 (s, 1H), 6.45 (t, *J* = 1.2 Hz, 1H), 6.34 (d, *J* = 6.8 Hz, 1H), 6.28 (t, *J* = 7.2 Hz, 1H), 5.37 (t, *J* = 5.2 Hz, 1H), 4.15–4.28 (m, 1H), 3.90–3.96 (m, 1H), 3.80–3.88 (m, 1H), 3.64–3.72 (m, 1H), 2.34 (s, 3H); HRMS (ESI) *m/z* 303.0785 [$(M+H)^+$, $C_{12}H_{13}F_2N_2O_5$ requires 303.0787].

Preparation of Pyrrolo-Gemcitabine 9

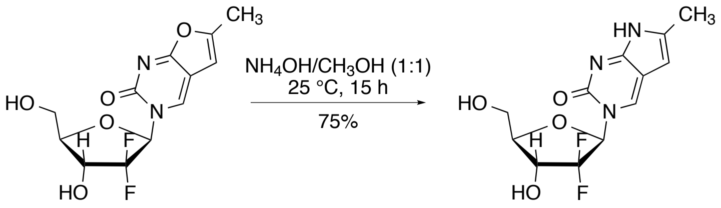

A solution of **8** (50 mg, 0.165 mmol) in MeOH (1.5 mL) was treated with NH_4OH (1.5 mL) at 25 ºC. After stirring for 15 h at the same temperature, the resulting mixture was concentrated *in vacuo*. The residue was purified by column chromatography (SiO_2, $CHCl_3$/MeOH = 8/1) to afford pyrrolo-gemcitabine **9** (37 mg, 75%) as a brown oil: ^{1}H NMR (400 MHz, DMSO-d_6) δ 11.24 (s, 1H) 8.37 (s, 1H), 6.33 (t, *J* = 7.2 Hz, 1H), 6.28 (d, *J* = 6.4 Hz, 1H), 5.92 (s, 1H), 5.34 (t, *J* = 5.2 Hz, 1H), 4.14–4.28 (m, 1H), 3.88 (dt, *J* = 8.8, 2.4 Hz, 1H), 3.80–3.86 (m, 1H), 3.64–3.71 (m, 1H), 2.21 (s, 3H); HRMS (ESI) *m/z* 302.0946 [$(M+H)^+$, $C_{12}H_{14}F_2N_2O_4$ requires 302.0947].
